# Phenolic Profiling of Grapevine Leaves and the Protective Effect of a Polyphenol-Rich *Vitis amurensis* Extract on *Lactiplantibacillus plantarum* Under Oxidative Stress

**DOI:** 10.3390/antiox15101266

**Published:** 2026-10-01

**Authors:** László Kőrösi, Okba Hatem, Franco Röckel, György Schneider, Szilvia Papp

**Affiliations:** 1 Research Institute for Viticulture and Oenology, University of Pécs, 7634 Pécs, Hungary; 2 Cochrane Hungary, Medical School, University of Pécs, 7624 Pécs, Hungary; okba.hatem@aok.pte.hu; 3 Julius Kühn Institute (JKI)—Federal Research Centre for Cultivated Plants, Institute for Grapevine Breeding Geilweilerhof, 76833 Siebeldingen, Germany; franco.roeckel@julius-kuehn.de; 4 Department of Medical Microbiology and Immunology, Medical School, University of Pécs, 7624 Pécs, Hungary; schneider.gyorgy@pte.hu; 5 Department of Biochemistry and Medical Chemistry, Medical School, University of Pécs, 7624 Pécs, Hungary; szilvia.papp.korosine@aok.pte.hu

**Keywords:** polyphenols, wild *Vitis* species, *Vitis vinifera*, grapevine leaf, flavonoids, quercetin, kaempferol, microbiome, gut microbiota, *Lactiplantibacillus plantarum*

## Abstract

Polyphenols are plant secondary metabolites widely recognized for their antioxidant properties and potential health benefits. Grapevine leaves are a rich yet underexplored source of these bioactive compounds. In this study, caftaric acid, and predominant flavonol glycosides were characterized in leaves of seventeen grapevine genotypes by high-performance liquid chromatography, while total phenolic content (TPC) was determined using the Folin–Ciocalteu assay. In addition, the growth-promoting effect of a polyphenol-rich *Vitis amurensis* leaf extract on *Lactiplantibacillus plantarum* and its protective effect under H_2_O_2_-induced oxidative stress were evaluated. Both TPC and phenolic composition were strongly genotype-dependent, with TPC ranging from approximately 20 to 100 mg gallic acid equivalents (GAE) g^−1^ dry weight (DW). Quercetin and kaempferol glycosides were the predominant flavonols, and quercetin-3-*O*-glucuronide, one of the most abundant derivatives, varied nearly tenfold among genotypes (approximately 2100–20,000 mg kg^−1^ DW). The leaf extract of *V. amurensis*, the genotype with the highest TPC, significantly promoted the *in vitro* growth of *L. plantarum* in a dose-dependent manner, resulting in higher viable cell counts after 24 h. The extract also markedly enhanced bacterial survival under H_2_O_2_-induced oxidative stress. At 2 mM H_2_O_2_, no bacterial growth was detected in the absence of the extract, whereas extract-supplemented cultures maintained substantial growth and reached a viable cell count of 1.8 × 10^8^ colony-forming units (CFU) mL^−1^ after 24 h. The phenolic patterns identified across the investigated genotypes provide a basis for selecting *Vitis* genotypes as sources of specific flavonol glycosides. Furthermore, our microbiological results provide preliminary evidence of the beneficial effects of *V. amurensis* leaf extract on *L. plantarum* and warrant further investigation of its potential functional applications.

## 1. Introduction

Polyphenols are a diverse group of secondary plant metabolites that contribute to the sensorial characteristics and oxidative stability of the plant, as well as protecting plant from UV radiation and pathogenic organisms [1]. These compounds are well-known for their potent biological properties and wide-ranging beneficial effects on human health, including antioxidant, anticancer, antimicrobial, anti-inflammatory and antiaging activities [2,3]. Their role in mitigating oxidative stress and inflammatory pathways that are involved in chronic degenerative diseases has been extensively documented [4]. Furthermore, recent research has revealed that these compounds positively influence gut microbiota, leading to additional health benefits for the population [5]. The variation in these beneficial activities is determined by the structure of the compound, which in turn depends on the source plant [6]. In this context, grapevines emerge as a rich source of phenolic compounds, encompassing a wide array of structurally diverse classes.

Grapevine (*Vitis* spp.) has been the subject of numerous phytochemical studies, particularly focusing on its berries. This attention is largely due to the global prominence of grapes as one of the most widely cultivated and consumed fruits, with worldwide production exceeding 74 million tons in 2022, according to recent data from the Food and Agriculture Organization of the United Nations [7]. Additionally, grape juice and seed extracts have garnered significant attention from researchers owing to the health benefits associated with grape-derived polyphenols [8]. However, recent studies have increasingly highlighted that other plant organs, such as leaves [9] and stems [10], constitute significant reservoirs of polyphenolic compounds. These vegetative wastes are generated in considerable amounts during green pruning and other canopy-management practices performed throughout the growing season to improve grape quality, with grapevine leaves constituting the main by-product [11]. Total fresh leaf biomass of 6.1–8.8 t ha^−1^ has been reported across different grapevine cultivars [12]. Experimental defoliation studies have shown reductions of approximately 30–40% in total vine leaf area [13,14]. Assuming that the removed leaves represent a comparable proportion of fresh leaf biomass, defoliation alone could therefore generate approximately 2–3.5 t fresh weight (FW) ha^−1^ of leaf material. Therefore, the utilization of leaves as a rich source of bioactive compounds could also contribute to the sustainable production of grapes, supporting the European Union and United Nations sustainability goals for a circular economy [15].

Grapevine leaves represent a fundamental element of traditional cuisine in several countries, particularly in the Mediterranean regions, where they are commonly stuffed and cooked, either fresh or preserved in brine [16]. *Vitis vinifera* (*V. vinifera*) leaves have long been used in European traditional medicine for their astringent and hemostatic properties, particularly in treating conditions such as diarrhea, bleeding, hemorrhoids, varicose veins, and other venous and circulatory disorders. In Turkish folk practices, the leaves are recognized for their diuretic properties. Moreover, infusions prepared from the leaves of *V. labrusca* have traditionally been used for hepatic and gastrointestinal disorders, diarrhea, fever, rheumatism, and headache [17].

The functional potential of grape leaf polyphenols may also extend to their ability to selectively modulate the growth and metabolic activity of probiotic microorganisms [18]. Among these, lactic acid bacteria, particularly *Lactiplantibacillus plantarum* (*L. plantarum*, formerly *Lactobacillus plantarum*), has attracted considerable attention due to its probiotic properties and metabolic versatility [19]. Unlike many lactobacilli that are restricted to specific environments, *L. plantarum* thrives in a wide range of ecological niches, including fermented dairy products, vegetables, meat, cereals, beverages, and the human gastrointestinal and urogenital tract [20]. This adaptability reflects its extensive metabolic capacity and genetic diversity, enabling the species to survive under varying environmental conditions while contributing to the fermentation, preservation, and quality of numerous food products [21].

*L. plantarum* benefits extend beyond food applications, as this probiotic species is essential for maintaining intestinal microbial homeostasis. The gut microbiota is a complex and dynamic microbial ecosystem that plays a vital role in human health by regulating food metabolism, immunological function, intestinal barrier integrity, and protection against pathogenic microorganisms [22]. In fact, dysregulation of this microbial system, known as dysbiosis, has been associated with various metabolic, inflammatory, and gastrointestinal disorders [23]. Therefore, interactions between dietary polyphenols and *L. plantarum* are of particular interest [24], given the ability of this species to survive gastrointestinal conditions, compete with opportunistic pathogens, modulate immune response and influence microbial composition [25,26].

In this work, we studied the total phenolic content and polyphenol composition of grapevine leaves from 17 diverse grapevine genotypes. Using high-performance liquid chromatography (HPLC), we focused on major phenolic constituents, especially caftaric acid and flavonol glycosides. The first objective was to compare the total phenolic content and detailed phenolic profiles of grapevine leaves collected from a diverse grapevine germplasm collection. The inclusion of both wild *Vitis* and *V. vinifera* enabled comparisons across closely and distantly related genotypes, allowing us to evaluate genotype-dependent differences in phenolic composition within a broader phylogenetic context. The second objective was to investigate the growth-promoting effect of a polyphenol-rich *V. amurensis* leaf extract on *L. plantarum* and its protective effect against H_2_O_2_-induced oxidative stress. The novelty of the present study lies in integrating comparative phenolic profiling across a diverse set of grapevine genotypes with the microbiological evaluation of a selected polyphenol-rich leaf extract, thereby linking genotype-dependent phytochemical diversity with a potential functional utilization of grapevine leaves.

## 2. Materials and Methods

### 2.1. Chemicals and Reagents

Acetonitrile and methanol (Promochem Optigrade, LGC Standards GmbH, Wesel, Germany) were ≥99.9%, gradient grade, suitable for HPLC. Caftaric acid and the following flavonol glycoside standards were purchased from Extrasynthese (Genay, France): quercetin-3-*O*-galactoside, quercetin-3-*O*-glucoside, quercetin-3-*O*-glucuronide, kaempferol-3-*O*-rutinoside, kaempferol-3-*O*-glucoside, and kaempferol-3-*O*-glucuronide. Glacial acetic acid (≥99.9%) and ethanol were purchased from Molar Chemicals Ltd., Halásztelek, Hungary. Folin–Ciocalteu′s phenol reagent, sodium carbonate (≥99.5%), gallic acid (≥98.0%), and 30% (*w*/*w*) hydrogen peroxide (H_2_O_2_) were purchased from Merck Life Science Kft., Budapest, Hungary. High-purity deionized water was obtained using a LaboStar 7 TWF-UV ultrapure water system (SG Wasseraufbereitung und Regenerierstation mbH, Barsbüttel, Germany).

### 2.2. Experimental Site and Plant Materials

Leaf samples were collected from the University of Pécs grapevine field germplasm collection, located on the southern slopes of the Mecsek Hills, Hungary (46°04′ N, 18°11′ E; 150 m a.s.l.). The selected grapevine genotypes included in this study were the follows: wild *Vitis* spp.: *V. amurensis*, *V. coignetiae*, *V. davidii*, *V. pentagona*, *V. piasezkii*, Riparia Gloire de Montpellier, *V. romanetii*, Rupestris du Lot, *V. sylvestris*; *V. vinifera* spp.: Dabouki, Furmint, Kishmish Vatkana, Manseng Petit Blanc, Pinot noir, Sárga muskotály, Tannat; Interspecific hybrid: Isabella (*V. Labrusca* × *V. vinifera*). Vines were grafted onto the rootstock ‘T5C’ and grown under non-irrigated open-field conditions. For each genotype, five vines were selected, with each vine representing one independent biological replicate. From each vine, the fully expanded healthy leaves were collected from randomly selected shoots at the fourth or fifth node counted from the base of the shoot, and then pooled to obtain the corresponding biological replicate. Thus, five independent biological replicates (*n* = 5) were prepared for each genotype. Samples were immediately frozen in liquid nitrogen in the field, transported to the laboratory, and then freeze-dried for 24 h using a ScanVac CoolSafe 110-4 Freeze Dryer (LaboGene ApS, Allerød, Denmark). The freeze-dried leaf samples were ground to a fine powder using an electric blade grinder prior to extraction.

### 2.3. Sample Preparation

#### 2.3.1. Preparation of Freeze-Dried Leaf Powder for Phenolics Analysis

Phenolic compounds were extracted from freeze-dried grapevine leaves according to a previously described method [27], with minor modifications. Freeze-dried leaf powder (25 mg) was suspended in 1 mL of 70% (*v*/*v*) ethanol and sonicated for 15 min in an ultrasonic bath (Elma Transsonic T 460, 35 kHz; Elma, Singen/Hohentwiel, Germany). The extracts were subsequently centrifuged at 20,660× *g* for 5 min, and the supernatants were filtered through 0.22 µm polyethersulfone (PES) syringe filters (FilterBio^®^, Labex Ltd., Budapest, Hungary). The resulting extracts were used for both total phenolic content (TPC) determination and high-performance liquid chromatography analysis with diode array detection (HPLC-DAD).

#### 2.3.2. Preparation of *V. amurensis* Leaf Extract for Microbial Assays

A larger quantity of *V. amurensis* leaf crude extract for the microbial assays was prepared as follows. Four aliquots (2.5 g each) of ground, freeze-dried *V. amurensis* leaves were suspended in 40 mL of 70% (*v*/*v*) ethanol in 50 mL centrifuge tubes and sonicated for 60 min. The extraction was subsequently continued for an additional 12 h under continuous rotation using a PTR-60 vertical multifunction rotator (Grant Instruments Ltd., Cambridgeshire, UK). The resulting extracts were centrifuged, and the supernatants were concentrated under reduced pressure using a rotary evaporator and subsequently lyophilized. The dry crude extract was stored at −25 °C, and fresh extract solutions were prepared immediately before each microbiological experiment. For the microbial assays, 110 mg of the crude extract powder was dissolved in 1 mL of deionized water by sonication for 5 min. The reconstituted extract was centrifuged at 20,660× *g* and passed through a 0.22 µm PES syringe filter prior to use. No heat sterilization was applied. The absence of microbial contamination was routinely verified using bacteria-free extract controls and was confirmed by both optical density (OD) monitoring and viable cell-count measurements.

### 2.4. Genetic Fingerprinting of Grapevine Genotypes

Total DNA was extracted from dried young leaves using the DNeasy 96 Plant Mini Kit (QIAGEN, Düsseldorf, Germany) according to the manufacturer’s instructions. Samples were genotyped using the nine GrapeGen06 SSR markers VVS2, VVMD5, VVMD7, VVMD25, VVMD27, VVMD28, VVMD32, VrZAG62, and VrZAG79, as listed in the *Vitis* International Variety Catalog [28]. The obtained SSR profiles were compared with those of reference cultivars and individuals of wild *Vitis* species. PCR was performed using the 2× KAPA2G Fast PCR Kit (Düren, Germany). Final reaction mixtures (5 μL) contained 1 ng of DNA, 10 pmol of each primer, and master mix. Amplification was performed in three multiplex PCRs using a GeneAmp PCR System 9700 thermal cycler (Applied Biosystems, Schwerte, Germany), with an initial denaturation at 95 °C for 3 min, followed by 30 cycles of denaturation at 95 °C for 15 s, annealing at 60 °C for 30 s, and extension at 72 °C for 30 s, with a final extension at 72 °C for 5 min. For fragment-length determination, 1 μL of PCR product was analyzed by capillary electrophoresis using an ABI 3130xl Genetic Analyzer (Applied Biosystems, Life Technologies, Waltham, MA, USA). Data were analyzed using GeneMapper 4.0 software (Applied Biosystems, Life Technologies, Waltham, MA, USA), and allele sizes were determined in base pairs (bp) by comparison with an internal size standard. The SSR genotyping results for the grapevine accessions are summarized in Table S1.

### 2.5. Determination of Total Phenolic Content by Folin–Ciocalteu Assay

Total phenolic content (TPC) was determined using the Folin–Ciocalteu assay according to a previously published method [27], with minor modifications. The procedure used in the present study is briefly described below. All leaf extracts prepared as described in Section 2.3.1. were diluted 50-fold with 70% (*v*/*v*) ethanol. An aliquot of 100 µL was transferred into 2 mL microcentrifuge tubes, followed by the addition of 500 µL of 10-fold diluted Folin–Ciocalteu reagent and 500 µL of 6% (*w*/*v*) sodium carbonate solution. The reaction mixture was vortex-mixed after each addition and incubated in the dark for 90 min. Absorbance was measured at 760 nm using a UV–Vis spectrophotometer (Shimadzu UV-1800, Kyoto, Japan). Calibration was performed using gallic acid standard solutions with five concentration levels in the range 0.5–8.0 mg L^−1^. TPC was calculated using a gallic acid calibration curve (y = 0.1171x + 0.0097, R^2^ = 0.9993) and expressed as mg gallic acid equivalents per gram of dry weight (mg GAE g^−1^ DW).

### 2.6. High-Performance Liquid Chromatography Analysis (HPLC-DAD)

Phenolic compounds were analyzed by HPLC-DAD according to a previously published method [27], with minor modifications. The analytical conditions used in the present study are briefly described below. HPLC-DAD analysis was performed using a Shimadzu Prominence UFLC system (Shimadzu, Kyoto, Japan) equipped with an online degasser (DGU-20A5R), binary pump (LC-20AD), autosampler (SIL-20AC HC), column oven (CTO-20AC), and diode-array detector (SPD-M20A). Separation was achieved on a Kinetex^®^ XB-C18 column (2.6 µm, 100 Å, 100 × 4.6 mm; Phenomenex, Torrance, CA, USA). The mobile phase consisted of 0.25% (*v*/*v*) acetic acid in water (solvent A) and 0.25% (*v*/*v*) acetic acid in acetonitrile (solvent B). The gradient program was as follows: 0–3 min, 0% B; 3–10 min, 0–8% B; 10–12 min, 8–16% B; 12–30 min, 16% B; 30–31 min, 100% B; and 31–36 min, 0% B for column re-equilibration. The flow rate was 1.0 mL min^−1^, the column temperature was maintained at 25 °C, and the injection volume was 20 µL. Extracts prepared as described in Section 2.3.1 were analyzed. Caftaric acid and flavonol glycosides were quantified from chromatograms recorded at 330 and 350 nm, respectively. External calibration was performed using authentic reference standards. Calibration equations and method validation parameters, including linearity, limits of detection and quantification, precision, repeatability, and recovery, are provided in Table S2.

### 2.7. Microbial Experiments

#### 2.7.1. Isolation and Identification of *L. plantarum*

The *L. plantarum* strain used in this study was isolated from a commercially available probiotic dietary supplement (JutaVit Probium 7 + Inulin capsules containing *L. plantarum* UALp-05™; JuvaPharma Kft., Felsőpakony, Hungary). The contents of one capsule were suspended in 10 mL of lysogeny broth (LB) and streaked directly onto LB agar plates. Following aerobic incubation at 37 °C for 48 h, single colonies with different morphologies were selected from the LB agar plates and purified by repeated streaking. Species identification was performed by matrix-assisted laser desorption ionization–time of flight mass spectrometry (MALDI-TOF MS) using a VITEK MS Prime system (bioMérieux, Marcy-l’Étoile, France). The isolate was identified as *L. plantarum*, with best and second-best identification scores of 2.28 and 2.22, respectively. The identified *L. plantarum* strain was stored in LB broth containing 30% (*v*/*v*) glycerol at −80 °C until further use.

#### 2.7.2. Growth Kinetics of *L. plantarum*

Bacterial growth was investigated in the absence and presence of *V. amurensis* leaf extract. LB broth was used for growth-kinetic measurements because its optical clarity provided a suitable background for continuous spectrophotometric monitoring of bacterial growth. The isolated *L. plantarum* strain was streaked onto LB agar plates and incubated aerobically at 37 °C for 48 h. A single colony was inoculated into 5 mL of LB broth and cultured under aerobic conditions without shaking at 37 °C for 18 h. The bacterial suspension was then adjusted to an optical density of 0.2 at 630 nm using fresh LB broth.

Aliquots of 900 µL of the bacterial suspension were transferred into 2 mL microcentrifuge tubes and mixed with 100 µL of distilled water (control) or with 1, 5, 25, or 50 µL of (110 mg mL^−1^) *V. amurensis* leaf extract (prepared in 2.3.2.) supplemented with 99, 95, 75, or 50 µL of distilled water, respectively. The final extract concentrations were 0.11, 0.55, 2.75, and 5.50 mg mL^−1^. The compositions of the bacterial cultures and the corresponding treatment-specific background controls are summarized in Table S3. Subsequently, 200 µL of each suspension was transferred into individual wells of a sterile, non-treated 96-well tissue culture microplate (Sarstedt, Nümbrecht, Germany). Each treatment was prepared in triplicate. Growth kinetics were monitored continuously at 37 °C using a temperature-controlled AMR-100 multimode microplate reader (Allsheng, Hangzhou, China). Optical density was recorded at 630 nm every 15 min for 24 h, and each measurement was preceded by 5 s of orbital shaking.

To evaluate the effect of oxidative stress, bacterial cultures were supplemented with hydrogen peroxide (H_2_O_2_). Briefly, 900 µL of bacterial suspension was mixed with 50 µL of *V. amurensis* leaf extract to obtain a final extract concentration of 5.50 mg mL^−1^. Subsequently, distilled water or 5, 10, 25, or 50 µL of a 200 mM H_2_O_2_ solution was added, and the total volume was adjusted with distilled water to obtain final H_2_O_2_ concentrations of 0, 1, 2, 5, and 10 mM, respectively. Each treatment was prepared in triplicate, and growth kinetics were recorded under the same conditions described above.

For all growth experiments, growth kinetic parameters were calculated independently for each biological replicate from the background-corrected optical density at 630 nm (OD_630_) growth curves. Growth curves were corrected for treatment-specific background absorbance by subtracting, at each time point, the mean OD_630_ of three independently prepared bacteria-free technical replicates containing the same concentrations of extract and/or H_2_O_2_ as the corresponding bacterial cultures. For the untreated control, the mean OD_630_ of three independently prepared bacteria-free LB medium blanks containing the corresponding volume of water was subtracted. The maximum growth rate was determined as the slope of the linear regression fitted over a 2 h interval centered on the inflection point (±1 h), identified from the maximum of the first derivative of each growth curve. Lag time was estimated by extrapolating this regression line to the initial background-corrected OD_630_ value. The area under the curve (AUC) was calculated by integration of the background-corrected growth curves over the 0–24 h interval. Final OD_630_ was defined as the background-corrected OD_630_ measured at the final time point (24 h).

#### 2.7.3. Determination of Viable Cell Counts

Viable cell counts were determined at the beginning of the experiment (0 h) and after 24 h of incubation using the bacterial suspensions recovered from the microplate wells. Ten-fold serial dilutions were prepared from each culture in LB medium. Aliquots (10 µL) of each dilution were spotted onto the edge of LB agar plates and spread linearly to improve colony separation and facilitate enumeration. For cultures exposed to 5 and 10 mM H_2_O_2_, 100 µL aliquots were plated because of the low viable cell counts. Following aerobic incubation at 37 °C for 18 h, colonies were counted on plates containing 10–100 colonies. Viable cell counts were expressed as colony-forming units per milliliter (CFU mL^−1^). Each treatment was independently prepared in three biological replicates (*n* = 3), which were individually subjected to both growth-kinetic and viable cell-count measurements.

### 2.8. Statistical Analysis

Statistical analyses were performed using IBM SPSS Statistics version 26.0 (IBM Corp., Armonk, NY, USA). Descriptive statistics were calculated for all quantified phenolic compounds. TPC and HPLC-DAD data were obtained from three (*n* = 3) and five (*n* = 5) biological replicates per genotype, respectively. Differences in total phenolic content and individual phenolic compound concentrations among genotypes were evaluated using Welch’s one-way ANOVA followed by the Games–Howell post hoc test because the assumption of homogeneity of variances was not met. For Q-glr and K-glr, *V. romanetii* was excluded from the quantitative genotype comparison because these compounds were not detected in any of the five biological replicates, resulting in zero within-group variance.

Growth kinetic parameters and viable cell counts in the non-stressed cultures were analyzed using one-way ANOVA followed by Dunnett’s multiple-comparison test, with the untreated culture as the control. For the H_2_O_2_-stressed cultures, corresponding treatments with and without the extract were compared using Welch’s independent-samples t-test where quantitative values were available for both treatments. Viable cell counts were log_10_-transformed prior to statistical analysis. Samples for which viable cell counts were not detectable or not reliably quantifiable were excluded from the statistical analysis of viable cell counts. Microbiological experiments were performed using three biological replicates (*n* = 3). Differences and correlations were considered statistically significant at *p* < 0.05.

Hierarchical cluster analysis (HCA) and Pearson correlation analyis were performed in R version 4.6.1 (R Foundation for Statistical Computing, Vienna, Austria), using RStudio 2026.07.1+147 (Posit Software, PBC, Boston, MA, USA). Pearson’s correlation coefficients were calculated using the mean values of five biological replicates for each genotype (*n* = 17). HCA was likewise performed using genotype mean values. Prior to clustering, the data were standardized using Z-score transformation. Samples and variables were clustered using Ward’s minimum variance method with squared Euclidean distance. The Pearson correlation heatmap was generated using the ComplexHeatmap package (version 2.28.0), whereas hierarchical clustering heatmaps of genotypes and phenolic compounds were generated using the pheatmap package (version 1.0.13).

## 3. Results

### 3.1. Total Phenolic Content and HPLC-DAD Analysis of Grapevine Leaf Extracts

**Figure 1:**
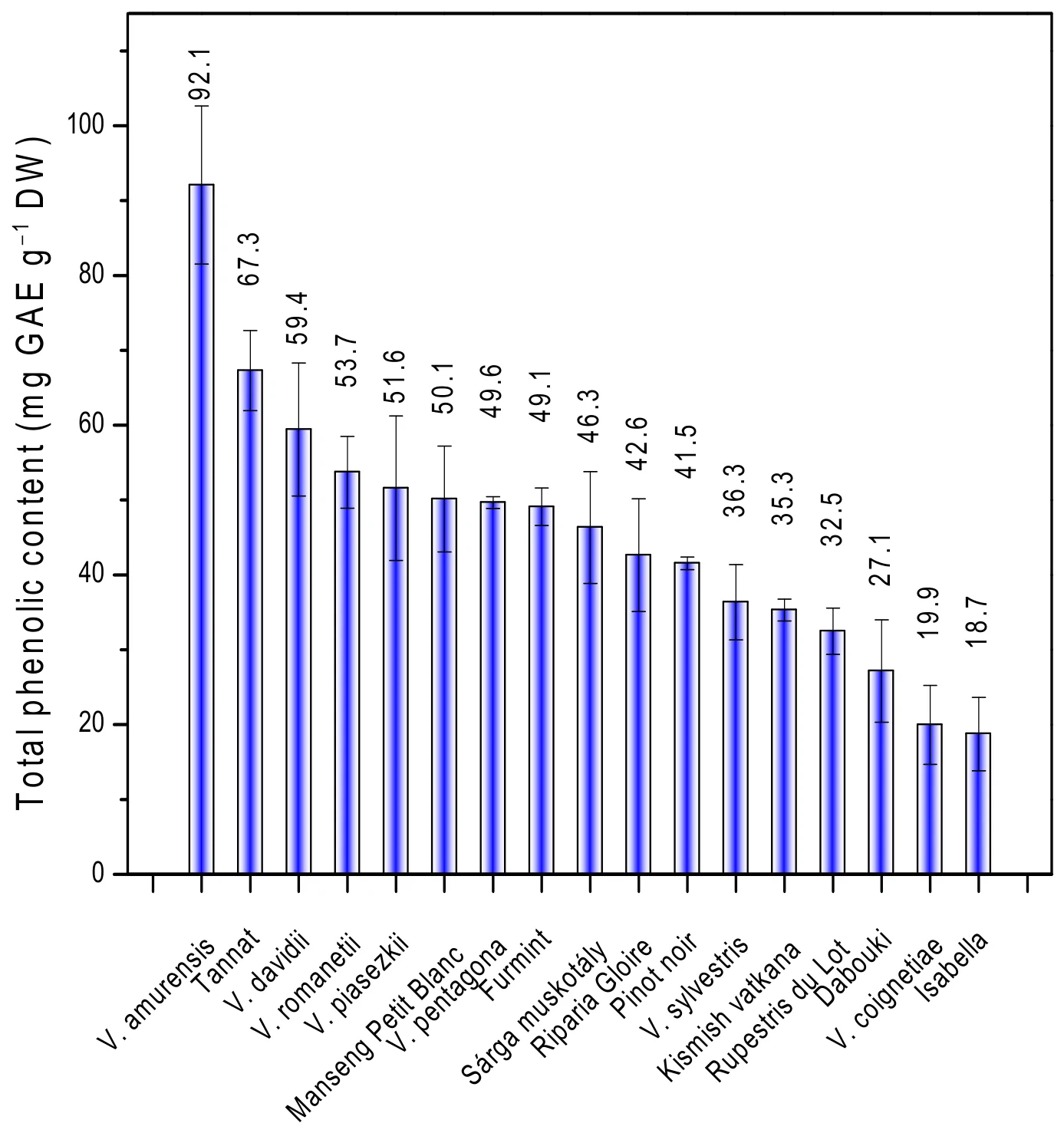
Variation in total phenolic content among 17 grapevine genotypes. Data are presented as mean ± SD (*n* = 3 biological replicates). Differences among genotypes were evaluated using Welch’s one-way ANOVA followed by the Games–Howell post hoc test (*p* < 0.05). Pairwise comparison results are provided in Table S4.

The total phenolic content (TPC) of the leaves from the 17 grapevine genotypes is shown in Figure 1. The TPC values varied widely, ranging from approximately 20 to 100 mg GAE g^−1^ DW. TPC differed significantly among the investigated grapevine genotypes (Welch’s ANOVA, *F*(16, 12.48) = 28.29, *p* < 0.001). Pairwise comparisons using the Games–Howell post hoc test revealed significant differences primarily between genotypes with relatively high and low TPC values (Table S4). *V. amurensis* exhibited an exceptionally high TPC, approaching 100 mg GAE g^−1^ DW. The TPC values of most genotypes (14 out of 17) ranged between approximately 30 and 70 mg GAE g^−1^ DW. *V. coignetiae* and ‘Isabella’ showed the lowest TPC values, both around 20 mg GAE g^−1^ DW.

**Figure 2:**
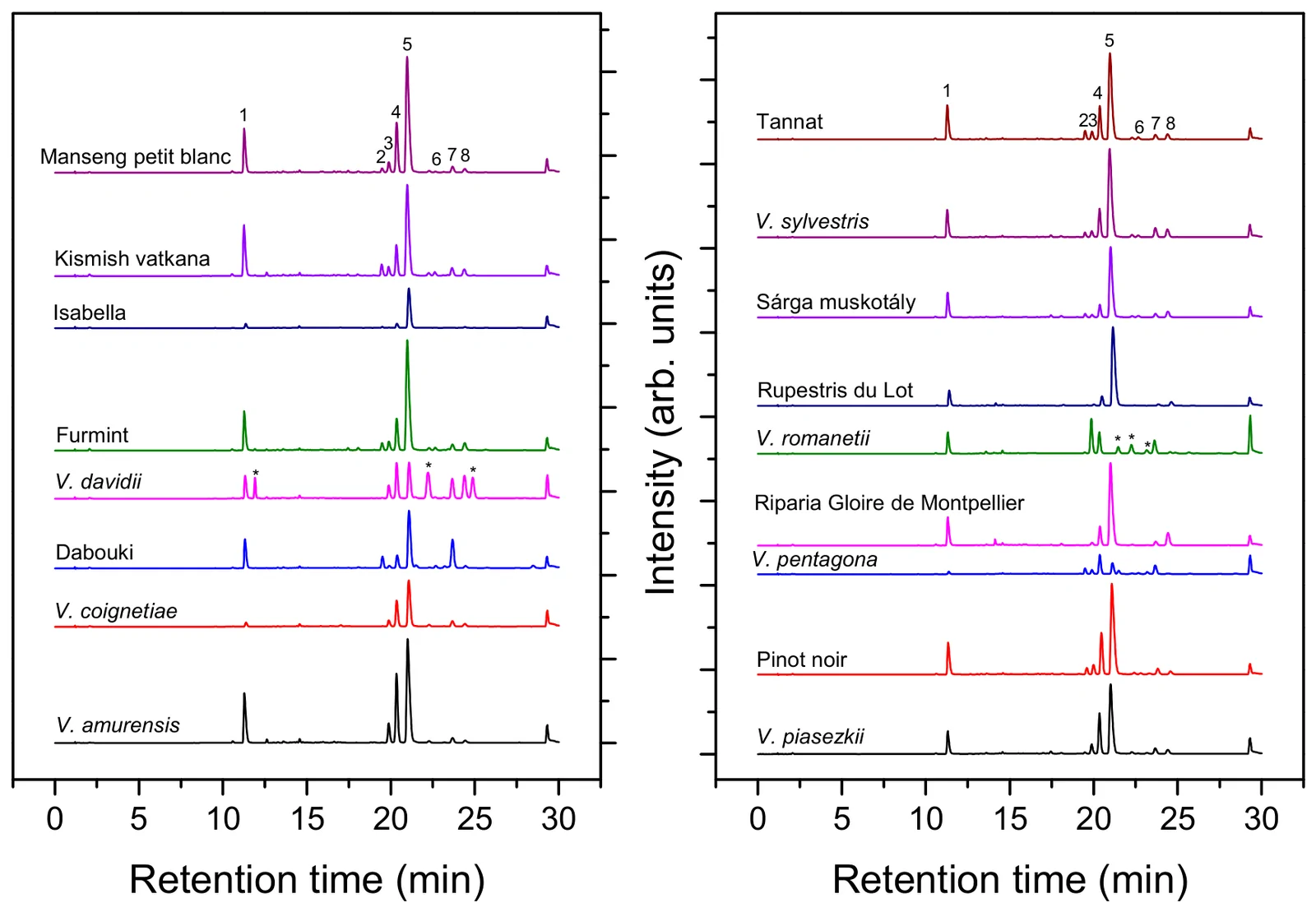
Comparison of HPLC-DAD chromatograms of leaf extracts from 17 *Vitis* genotypes recorded at 350 nm. 1: caftaric acid, 2: quercetin-3-*O*-rutinoside, 3: quercetin-3-*O*-galactoside, 4: quercetin-3-*O*-glucoside, 5: quercetin-3-*O*-glucuronide, 6: kaempferol-3-*O*-rutinoside, 7: kaempferol-3-*O*-glucoside, 8: kaempferol-3-*O*-glucuronide. Asterisks (*) indicate unidentified compounds.

Representative HPLC-DAD chromatograms of the 17 grapevine genotypes are depicted in Figure 2. Among the phenolic compounds, the hydroxycinnamic acid derivative caftaric acid, together with several flavonols corresponding to glycosylated derivatives of quercetin and kaempferol, were identified based on their retention times and UV–Vis spectra. In *V. davidii* and *V. romanetii*, several additional peaks were observed in the flavonol region of the chromatograms. Based on their UV–Vis spectral characteristics, which closely resembled those of the identified flavonol glycosides, these compounds were tentatively assigned to this phenolic group. In addition, *V. davidii* contained an unidentified compound with a UV–Vis spectrum highly similar to that of caftaric acid but with a different retention time (11.9 min).

**Figure 3:**
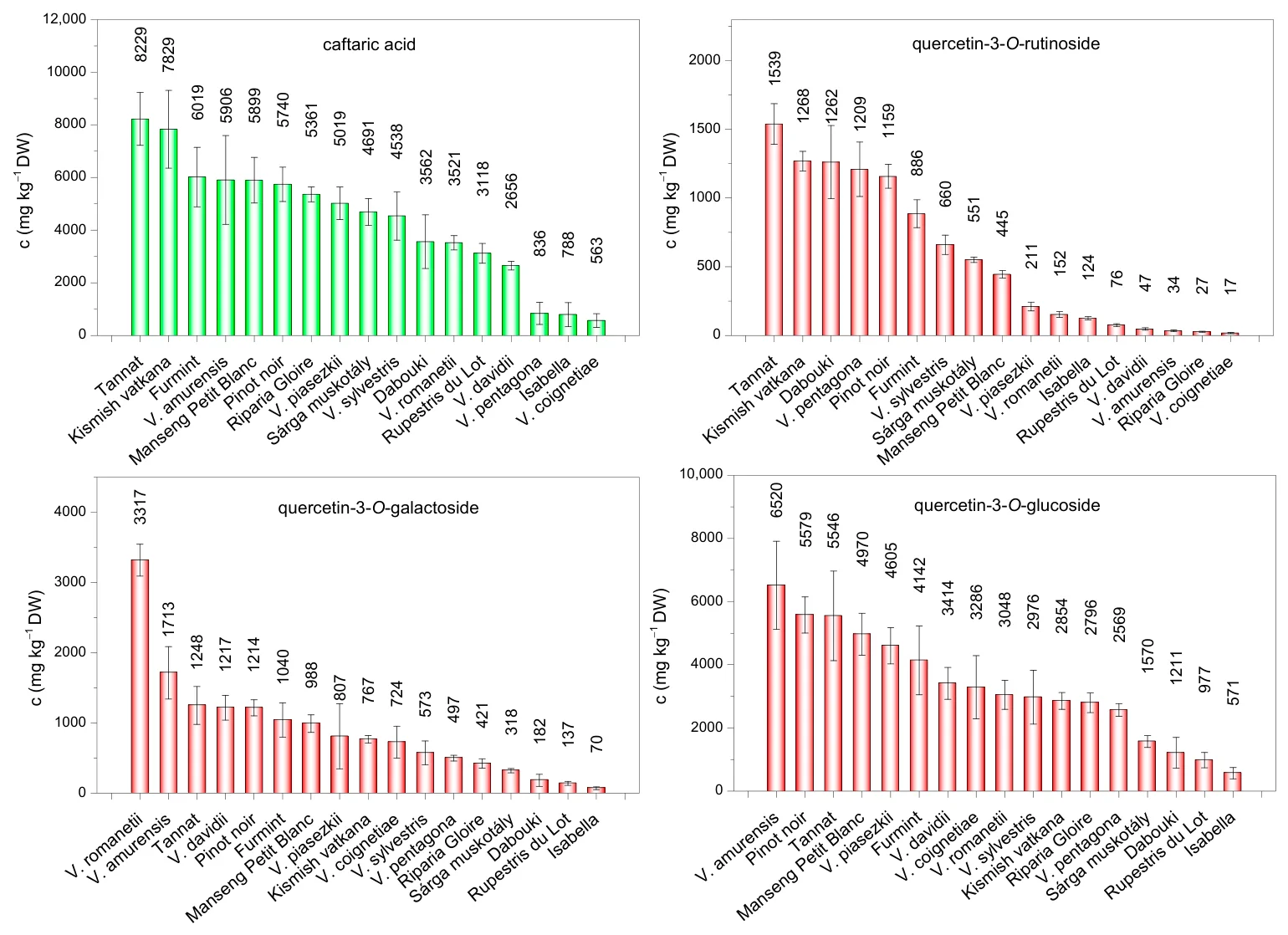
Genotype-dependent variation in caftaric acid, quercetin-3-*O*-rutinoside, quercetin-3-*O*-galactoside, and quercetin-3-*O*-glucoside contents in grapevine leaves. Data are presented as mean ± SD (*n* = 5 biological replicates). Differences among genotypes were evaluated separately for each phenolic compound using Welch’s one-way ANOVA followed by the Games–Howell post hoc test (*p* < 0.05). Pairwise comparison results are provided in Tables S5–S8.

**Figure 4:**
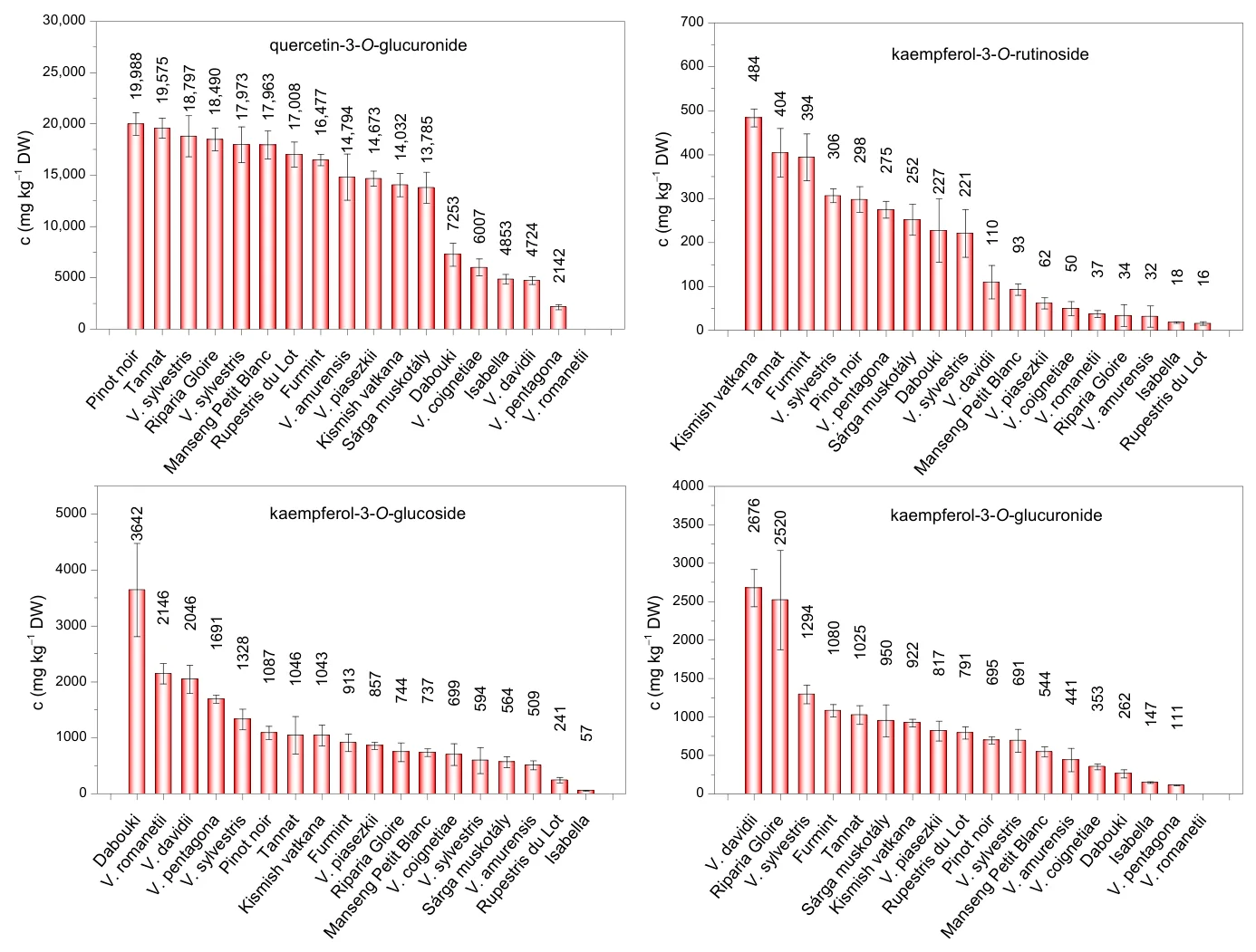
Genotype-dependent variation in quercetin-3-*O*-glucuronide, kaempferol-3-*O*-rutinoside, kaempferol-3-*O*-glucoside, kaempferol-3-*O*-glucuronide contents in grapevine leaves. Data are presented as mean ± SD (*n* = 5 biological replicates). Differences among genotypes were evaluated separately for each phenolic compound using Welch’s one-way ANOVA followed by the Games–Howell post hoc test (*p* < 0.05). Pairwise comparison results are provided in Tables S9–S12.

Figure 3 and Figure 4 show the concentrations of the major phenolic compounds in the leaves of the different grapevine genotypes. Significant genotype-dependent differences were observed for all quantified phenolic compounds, with the complete pairwise comparisons provided in Tables S5–S12. Caftaric acid (CA) occurred in the highest amounts in ‘Tannat’ (8229 mg kg^−1^ DW) and ‘Kishmish vatkana’ (7829 mg kg^−1^ DW). In contrast, *V. pentagona*, ‘Isabella’, and *V. coignetiae* contained approximately one-tenth of these levels. The CA content of the remaining genotypes ranged from approximately 2600 to 6000 mg kg^−1^ DW. Among the quercetin derivatives, quercetin-3-*O*-rutinoside (Q-rut) reached its highest concentration in ‘Tannat’ (1539 mg kg^−1^ DW), whereas *V. coignetiae* contained only 17 mg kg^−1^ DW. Low Q-rut levels were also observed in ‘Riparia Gloire de Montpellier’ (27 mg kg^−1^ DW), *V. amurensis* (34 mg kg^−1^ DW), Rupestris du Lot (76 mg kg^−1^ DW), ‘Isabella’ (124 mg kg^−1^ DW), *V. romanetii* (152 mg kg^−1^ DW), and *V. piasezkii* (211 mg kg^−1^ DW). Q-rut showed particularly pronounced genotype-dependent variation, with significant differences observed between a large proportion of the genotype pairs (Table S6). The concentration of quercetin-3-*O*-galactoside (Q-gal) ranged from 70 to 3317 mg kg^−1^ DW, with *V. romanetii* exhibiting the highest level. Depending on the genotype, quercetin-3-*O*-glucoside (Q-glc) varied between 571 and 6520 mg kg^−1^ DW, with *V. amurensis* showing the highest concentration. Quercetin-3-*O*-glucuronide (Q-glr) was the predominant flavonol glycoside in most genotypes, reaching concentrations between approximately 13,800 and 20,000 mg kg^−1^ DW in 12 of the 17 genotypes. Lower Q-glr levels (2100–7250 mg kg^−1^ DW) were observed in ‘Dabouki’, *V. coignetiae*, ‘Isabella’, *V. davidii*, and *V. pentagona*, while it was not detected in *V. romanetii*. These marked concentration differences were reflected in significant pairwise differences among numerous genotypes (Table S9). In general, the concentrations of kaempferol glycosides were lower than those of the quercetin glycosides. Kaempferol-3-*O*-rutinoside (K-rut) ranged from 16 to 484 mg kg^−1^ DW across the genotypes. ‘Dabouki’ exhibited an exceptionally high concentration of kaempferol-3-*O*-glucoside (K-glc) (3642 mg kg^−1^ DW), whereas the remaining genotypes contained between 57 and 2146 mg kg^−1^ DW. *V. davidii* and ‘Riparia Gloire de Montpellier’ showed the highest concentrations of kaempferol-3-*O*-glucuronide (K-glr) (approximately 2600 mg kg^−1^ DW), while the remaining genotypes ranged from approximately 100 to 1300 mg kg^−1^ DW. Similarly to Q-glr, K-glr was not detected in *V. romanetii*.

Values are presented as mean ± SD (*n* = 3). Viable cell counts at 24 h were determined by plate counting as described in Section 2.7.3 of the Materials and Methods. Statistical analyses of viable cell counts were performed on log_10_-transformed CFU mL^−1^ values. For non-stressed cultures, statistical significance was assessed by one-way ANOVA followed by Dunnett’s multiple-comparison test using the untreated culture as the control. Under oxidative stress, cultures exposed to H_2_O_2_ in the presence of the extract were compared with the corresponding H_2_O_2_-treated controls without extract using Welch’s independent-samples t-test. Asterisks indicate significant differences from the corresponding control: * *p* < 0.05, ** *p* < 0.01, and *** *p* < 0.001.

### 3.2. Effects of V. amurensis Leaf Extract on L. plantarum Growth and Survival Under Oxidative Stress

**Figure 5:**
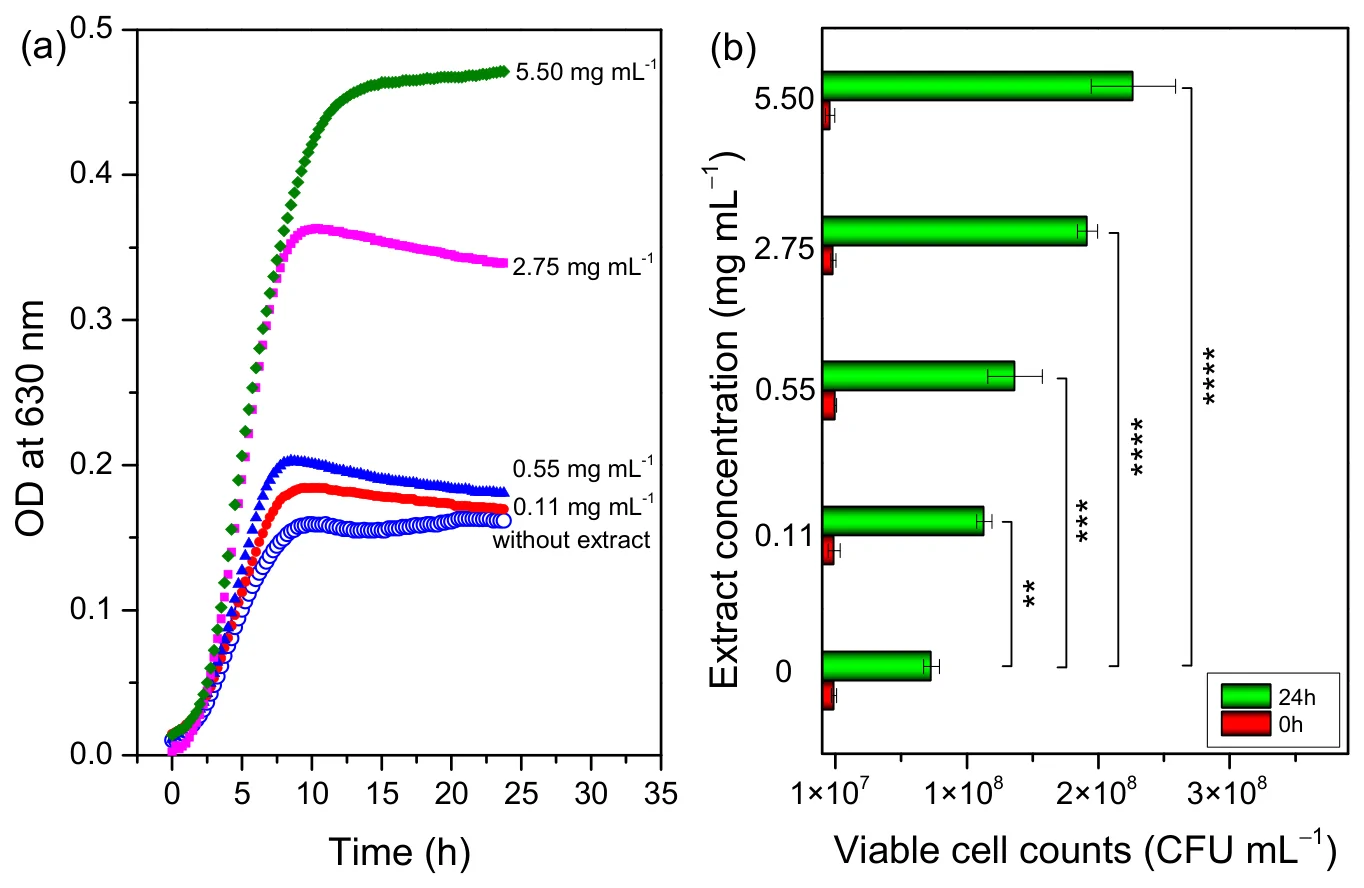
Growth-promoting effect of *V. amurensis* leaf extract on *L. plantarum*. (**a**) Background-corrected growth curves recorded in the presence of different concentrations of *V. amurensis* leaf extract. Background correction was performed using bacteria-free blanks containing the corresponding extract concentrations. (**b**) Corresponding viable cell counts determined at 0 and 24 h. Data are presented as mean ± SD (*n* = 3). Asterisks indicate significant differences from the untreated control according to Dunnett’s multiple-comparison test: ** *p* < 0.01, and *** *p* < 0.001, and **** *p* < 0.0001.

Figure 5a shows the growth curves of *L. plantarum* in the absence and the presence of *V. amurensis* extract at four different levels (0.11, 0.55, 2.75, and 5.50 mg mL^−1^). The phenolic composition of the crude extract used in the microbiological assays was verified by HPLC-DAD (Table S13). At the highest extract concentration (5.50 mg mL^−1^), CA and total flavonol glycosides were present in the culture medium at 407.1 ± 3.5 and 497.9 ± 2.2 mg L^−1^, respectively. Quantitative analysis of the growth curves showed a dose-dependent growth-promoting effect of the extract (Table 1). The maximum growth rate increased from 0.028 ± 0.002 OD_630_ h^−1^ in the untreated control to 0.068 ± 0.001 OD_630_ h^−1^ at 5.50 mg mL^−1^ extract (2.43-fold increase), while the AUC increased from 3.32 ± 0.11 OD_630_ × h to 8.55 ± 0.19 OD_630_ × h (2.58-fold increase). The lag time also increased with increasing extract concentration, from 1.62 ± 0.07 h in the untreated control to 2.28 ± 0.06 h at 5.50 mg mL^−1^ extract, indicating that bacterial adaptation to the extract-supplemented medium required a longer period before the onset of rapid growth. Significant changes in all growth kinetic parameters were observed from 0.55 mg mL^−1^, whereas the lowest extract concentration (0.11 mg mL^−1^) did not significantly affect these parameters. The growth-promoting effect was further supported by viable cell counts (Figure 5b; Table 1). The initial viable cell count was 9.0 × 10^6^ CFU mL^−1^ and increased to 8.3 × 10^7^ CFU mL^−1^ after 24 h in the control culture. In the presence of the extract, the viable cell counts after 24 h reached 1.2 × 10^8^, 1.5 × 10^8^, 2.0 × 10^8^, and 2.4 × 10^8^ CFU mL^−1^ at 0.11, 0.55, 2.75, and 5.50 mg mL^−1^ of extract, respectively, with significantly higher counts than the untreated control at all extract concentrations.

**Figure 6:**
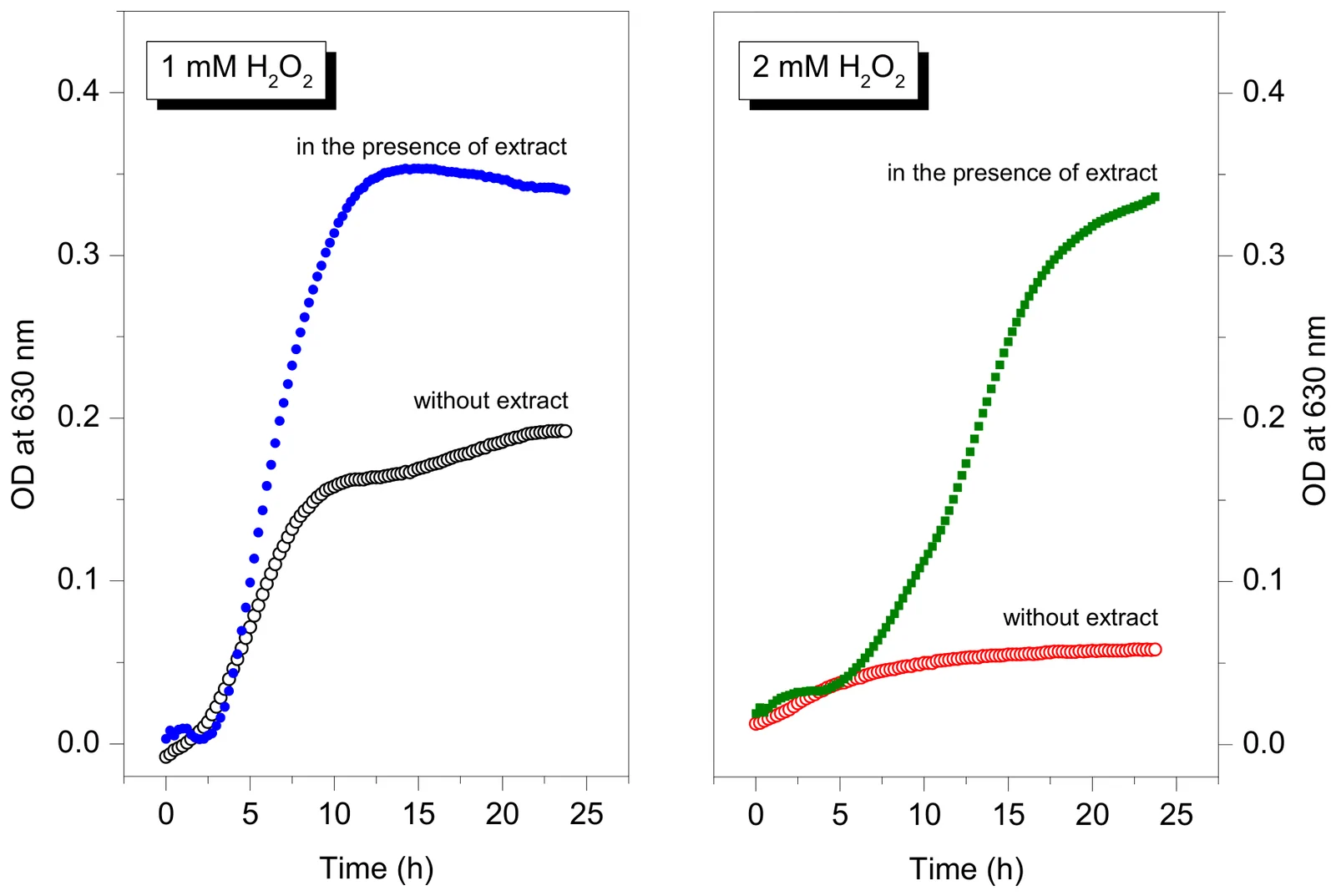
Protective effect of *V. amurensis* leaf extract on the growth of *L. plantarum* under H_2_O_2_-induced oxidative stress. Growth curves were recorded in the presence of 1 mM H_2_O_2_ (left panel) and 2 mM H_2_O_2_ (right panel), with or without *V. amurensis* leaf extract at a final concentration of 5.50 mg mL^−1^. Growth curves were corrected for treatment-specific background absorbance using bacteria-free blanks containing the corresponding concentrations of H_2_O_2_ and extract. Data are presented as mean (*n* = 3).

The growth-promoting effect of the *V. amurensis* extract was further investigated under H_2_O_2_-induced oxidative stress. Growth kinetic parameters and viable cell counts obtained in the presence of 1 and 2 mM H_2_O_2_, with or without 5.50 mg mL^−1^ extract, are summarized in Table 1, while the corresponding background-corrected growth curves are shown in Figure 6. At 1 mM H_2_O_2_, the extract significantly increased the maximum growth rate, AUC, final OD_630_, and viable cell count compared with the corresponding H_2_O_2_-treated culture without extract (Table 1). The maximum growth rate increased from 0.0264 ± 0.0017 to 0.0593 ± 0.0008 OD_630_ h^−1^, while the AUC increased from 3.15 ± 0.23 OD_630_ × h to 6.20 ± 0.84 OD_630_ × h. Similarly to the extract itself, H_2_O_2_ exposure also prolonged the lag phase, indicating that both treatments increased the time required for bacterial adaptation before rapid growth commenced. At 1 mM H_2_O_2_, the lag time increased from 1.62 ± 0.07 h in the untreated control to 1.97 ± 0.06 h, and was further prolonged to 3.37 ± 0.07 h when H_2_O_2_ and the extract were applied together. At 2 mM H_2_O_2_, no bacterial growth was detected in the absence of the extract, whereas cultures supplemented with 5.50 mg mL^−1^ extract showed measurable growth, reaching a final OD_630_ of 0.34 ± 0.02 and a viable cell count of 1.8 ± 0.2 × 10^8^ CFU mL^−1^ after 24 h (Figure 6; Table 1). Notably, the lag time in this system was prolonged to 4.97 ± 0.71 h.

The protective effect of the extract was further evaluated under severe oxidative stress using 5 and 10 mM H_2_O_2_ (Table 1). In the absence of the extract, no viable cells were detected after 24 h at either H_2_O_2_ concentration. In contrast, cultures supplemented with 5.50 mg mL^−1^ *V. amurensis* extract retained quantifiable viable cells at 5 mM H_2_O_2_ (5.5 ± 0.5 × 10^2^ CFU mL^−1^). At 10 mM H_2_O_2_, only sporadic colonies were recovered after 24 h and the viable cell number could therefore not be reliably quantified. These results demonstrate that the *V. amurensis* extract enhanced the survival of *L. plantarum* under H_2_O_2_-induced oxidative stress.

### 3.3. Hierarchical Cluster and Pearson Correlation Analyses

**Figure 7:**
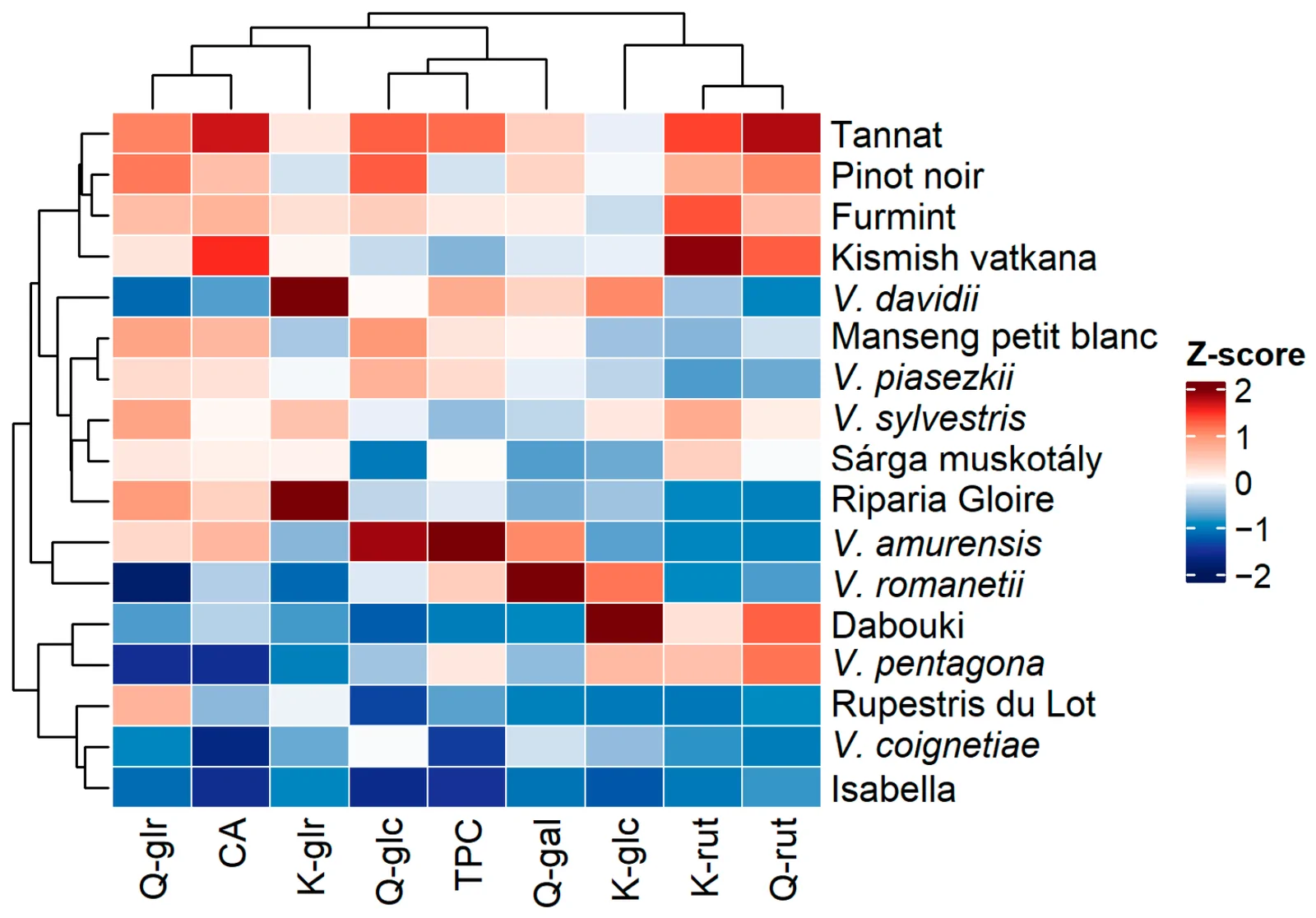
Hierarchical cluster analysis and heatmap based on TPC and individual phenolic compound concentrations in leaves of 17 grapevine genotypes.

Hierarchical cluster analysis of the genotype mean values resolved the 17 grapevine genotypes into three major clusters (Figure 7). The heatmap revealed marked differences in the relative abundance of the investigated phenolic compounds among genotypes. ‘Isabella’, *V. coignetiae*, and ‘Rupestris du Lot’ were characterized predominantly by below-average concentrations of the investigated compounds, whereas ‘Tannat’ showed above-average concentrations of most phenolic compounds.

**Figure 8:**
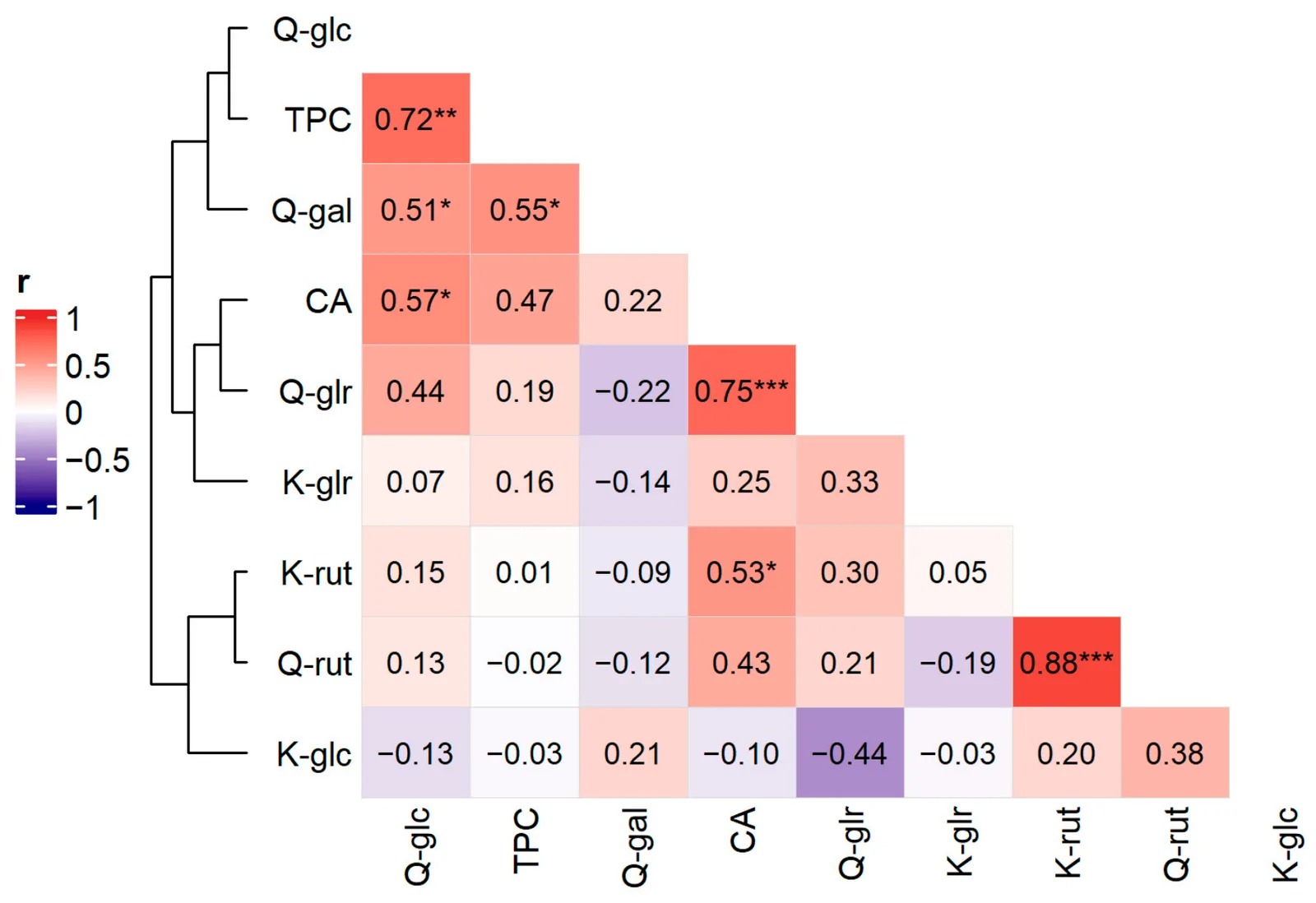
Pearson correlation heatmap with hierarchical clustering of TPC and individual phenolic compounds in grapevine leaves (*n* = 17 genotypes). Pearson’s correlation coefficients (*r*) are shown in the cells. Significance levels for Pearson correlations: * *p* < 0.05, ** *p* < 0.01, and *** *p* < 0.001.

Pearson correlation analysis revealed several significant positive associations among TPC and individual phenolic compounds (Figure 8). The strongest positive correlation was observed between K-rut and Q-rut (*r* = 0.880, *p* < 0.001), followed by CA and Q-glr (*r* = 0.749, *p* < 0.001). TPC was positively correlated with Q-glc (*r* = 0.722, *p* < 0.01) and Q-gal (*r* = 0.554, *p* < 0.05). Significant positive correlations were also observed between CA and Q-glc (*r* = 0.576, *p* < 0.05) and between Q-glc and Q-gal (*r* = 0.512, *p* < 0.05).

## 4. Discussion

Our results demonstrated that the total phenolic content (TPC) of leaves varied significantly among the investigated *Vitis* genotypes, ranging from approximately 20 to 100 mg GAE g^−1^ DW. For example, the TPC of the interspecific hybrid ‘Isabella’ (*V. labrusca* × *V. vinifera*) was only about one-fifth of that determined in the leaves of *V. amurensis*. Notably, *V. amurensis* exhibited the highest TPC among all investigated genotypes, exceeding the mean total phenolic contents previously reported for both the berry seeds (87.5 ± 0.7 mg GAE g^−1^ DW) and skins (44.7 ± 2.9 mg GAE g^−1^ DW) of a polyphenol-rich grape cultivar Cabernet Sauvignon (*V. vinifera*) [29]. These findings demonstrate that grapevine leaves are not only a rich source of phenolic compounds but, in selected genotypes, they may contain even higher total phenolic contents than grape berries themselves.

Despite their high phenolic content, grape leaves remain a relatively underutilized as a food ingredient [30], largely because they have considerably less consumer appeal than grape berries. Consequently, their culinary use is largely confined to a limited range of traditional dishes. Among the best-known examples are waraq enab, commonly consumed in middle Eastern countries, such as Syria, and dolma, which features in Turkish and other regional culinary traditions. These preparations typically compromise grapevine leaves filled with rice, vegetables, and, in some variants, meat [31,32]. The widespread culinary use of grape leaves in Mediterranean and Middle Eastern cuisines has also stimulated research into their preservation methods and postharvest shelf life [33]. As an underutilized by-product, grape leaves are increasingly attracting attention as a potential source of bioactive compounds for functional food applications due to their high polyphenol content and antioxidant activity [30]. Recently, *V. vinifera* leaf extracts have been encapsulated into nanovesicles and incorporated into kefir, significantly improving the antioxidant capacity of the final product [34]. Moreover, it was demonstrated that micromixer-assisted poly(3-hydroxybutyrate-co-3-valerate) microencapsulation was an effective and potentially safe method for protecting and delivering the polyphenols in *V. vinifera* leaf extract [35]. In addition, faba bean protein isolate-grape leaf polyphenol conjugates have recently been proposed as functional component in foods [36]. Grape leaf extracts have also been shown to alleviate high-fat diet-induced obesity in mice through inhibition of pancreatic lipase activity [37].

Although TPC provides a useful estimate of overall phenolic abundance, it does not reflect the qualitative differences in phenolic composition among genotypes. To gain deeper insight into genotype-dependent phenolic diversity, the individual phenolic constituents were characterized by HPLC-DAD. The chromatographic analyses revealed that CA together with flavonol glycosides constituted the predominant phenolic constituents of grapevine leaves in both wild *Vitis* species and *V. vinifera* cultivars (Figure 2). Both the qualitative composition and the abundance of individual phenolic compounds proved to be strongly genotype-dependent. The concentrations of several flavonol glycosides varied by as much as two orders of magnitude among the investigated genotypes, whereas even CA and Q-glr differed by approximately one order of magnitude (Figure 3 and Figure 4). Interestingly, these genotype-dependent differences were not restricted to compound concentrations but also involved distinct flavonol glycosylation patterns, indicating differences in flavonoid biosynthesis among grapevine genotypes.

Overall, hierarchical clustering resolved three major genotype clusters (Figure 7), while the accompanying heatmap highlighted marked differences in the relative abundance of phenolic compounds among the investigated genotypes. As expected based on the TPC results, the ‘Isabella’ cultivar contained the lowest amounts of several phenolic compounds among the investigated genotypes. The HCA heatmap revealed that the leaves of ‘Isabella’, *V. coignetiae*, and ‘Rupestris du Lot’ were characterized by the highest proportion of phenolic compounds occurring below the overall mean concentration. In contrast, the leaves of ‘Tannat’ were characterized by above-average concentrations of most phenolic compounds. Given its exceptionally high TPC, it was unexpected that *V. amurensis* exceeded the other genotypes only in Q-glc content, whereas the concentrations of kaempferol derivatives and Q-rut remained below the overall mean. This observation suggests that the exceptionally high TPC of *V. amurensis* cannot be explained solely by the quantified flavonol glycosides and caftaric acid. It should also be considered that the Folin–Ciocalteu assay is not specific to phenolic compounds and may respond to other reducing constituents. Thus, additional phenolic compounds and/or other reducing constituents that were not quantified in the present study may contribute to the high TPC observed for *V. amurensis*. Further studies will be required to identify the compounds responsible for this discrepancy.

It should also be highlighted that one sub-cluster included ‘Tannat’, ‘Pinot noir’, ‘Furmint’, and ‘Kismish Vatkana’, which is consistent with their genetic relationships, as they all belong to *V. vinifera*. However, it was interesting that ‘Dabouki’, which also belongs to *V. vinifera*, was clustered with a wild species, *V*. *pentagona*. The remarkably ancient ‘Sárga muskotály’ cultivar and the wild *V*. *sylvestris* are also located in a separate sub-cluster, whose close evolutionary and genetic relationship has already been clearly verified by previous genomic studies [38,39,40,41]. The close relationship between these two genotypes could also be inferred solely from their phenolic fingerprints, demonstrating that chemotaxonomic profiling is capable of capturing evolutionary relationships previously established by molecular genetic studies. Another separate sub-cluster included *V. amurensis* and *V*. *romanetii*; although both possess a unique phenolic pattern, Q-glc was present in an outstandingly high concentration in *V. amurensis*, while Q-gal was similarly high in *V. romanetii*.

Although the phenolic pattern defined by CA and flavonol glycosides is relatively simple, it provides a characteristic chemical fingerprint for each grapevine genotype. Pearson correlation analysis (Figure 8) revealed several significant correlations among the major phenolic compounds, indicating that the concentrations of these metabolites co-varied across the investigated genotypes. For instance, a strong positive correlation was found between CA and Q-glr, as well as between Q-rut and K-rut. The observed correlations among individual phenolic compounds suggest coordinated regulation within the phenylpropanoid pathway, particularly in the branch leading to flavonol biosynthesis. Flavonoids, including flavonols, are synthesized through the phenylpropanoid pathway, and numerous MYB transcription factors regulating this pathway have been identified in grapevine [42,43]. Although the biosynthesis of flavonol aglycones has been extensively investigated, considerably less is known about the regulation of flavonol glycosylation and the mechanisms controlling genotype-dependent accumulation of individual glycosides.

Strong positive correlations were also observed between Q-glc and TPC, and between Q-gal and TPC. Overall, the Pearson correlation analysis supported the HCA results, as strongly positively correlated phenolic compounds were generally grouped within the same clusters on the dendrograms (Figure 7). Among these relationships, the particularly strong association between Q-glc and TPC, together with the exceptionally high Q-glc concentration detected in the leaves of *V. amurensis*, suggests that this flavonol glycoside is one of the major contributor to the reducing capacity measured by the Folin–Ciocalteu assay.

Beyond their biological significance, the phenolic fingerprints obtained in this study are also valuable not only for improving our understanding of genetic relationships among grapevine genotypes but also from a chemotaxonomic perspective. Such phytochemical information facilitates the identification of genotype-specific sources of individual phenolic compounds and thereby supports their targeted extraction and isolation from plant materials. The present dataset may serve as a phytochemical map revealing both the qualitative and quantitative distribution of phenolic constituents, thereby enabling the targeted selection of grapevine genotypes enriched in specific phenolic compounds. Furthermore, the analytical dataset presented here may provide a useful resource for future investigations in which the extraction, purification, and isolation of specific phenolic compounds constitute essential objectives. Since the isolation of polyphenols from plant-derived matrices is often challenging because of their structural diversity and the complexity of plant extracts [44,45], prior knowledge of genotype-specific phenolic composition may substantially improve the efficiency of such studies.

Our results demonstrate that grapevine leaves can be exceptionally rich sources of flavonols. Depending on the genotype, their total flavonol content—present in the form of glycosylated derivatives—can exceed 30,000 mg kg^−1^ DW, corresponding to more than 3% of the leaf dry weight. Quercetin- and kaempferol glycosides were the most abundant flavonols detected in grapevine leaves. Among quercetin glycosides, Q-glr was one of the most prevalent forms (Figure 4) and, being considerably more water-soluble than the corresponding aglycone, has attracted increasing attention for its potential health benefits and food applications [46,47]. Additionally, the antimicrobial [48] and microbiota-modulating effects [49] of quercetin have been extensively investigated. Owing to the complexity of plant-derived extracts, it is very difficult, if not impossible, to predict their microbiological effects. Consequently, an empirical approach is always necessary to find extracts with the desired microbiological effect. To investigate the effect of grapevine leaf extract on *L. plantarum*, we selected *V. amurensis*, the genotype with the highest TPC. The results showed that *V. amurensis* leaf extract significantly promoted the growth of *L. plantarum* (Figure 5a and Table 1). This growth-promoting effect was dose-dependent, with increasing extract concentrations resulting in progressively enhanced bacterial growth and higher viable cell counts after 24 h. Our results are consistent with previously published data, according to which a flavonol glycoside (Q-rut) promoted the growth of *Lactobacillus* sp. [50]. This suggests that flavonol glycosides present in the *V. amurensis* extract may contribute to the observed growth-promoting effect. Nevertheless, because the crude extract represents a complex mixture, the contribution of non-phenolic constituents to the observed growth-promoting effect cannot be excluded.

A recently published study demonstrated that *L. plantarum* PBS067 was able to grow in the presence of grape leaf (*V. vinifera* L.) extract without toxic effects [51]. This finding is consistent with our results and provides further evidence for a beneficial effect of grape leaf extracts on the growth of *L. plantarum*. On the other hand, Wang et al. found that quercetin inhibited the *in vitro* growth of *E. coli* and *S. aureus* by damaging the bacterial cell wall and cell membrane, leading to increased permeability of these structures. In addition to its antibacterial effects against pathogenic bacteria, quercetin was shown to increase the abundance of *Lactobacillus* and *Bifidobacterium* in the cecal microbiota of broiler chickens in vivo [48]. Interestingly, a previous study reported that quercetin exhibited an inhibitory effect, which was particularly strong against *Ruminococcus gauvreauii*, *Bacteroides galacturonicus*, and *Lactobacillus* sp. [50]. However, rutin (Q-rut), the glycosylated form of quercetin, showed no inhibitory effect on intestinal bacteria and even slightly promoted the growth of *Lactobacillus* sp. [50]. Milutinović et al. found that extracts prepared from various polyphenol-rich medicinal plants, including *Gentiana asclepiadea* L., *Hypericum perforatum* L., *Satureja montana* L., and *Achillea millefolium* L., promoted the growth of *L. plantarum* as well as a probiotic yeast, *Saccharomyces boulardii* [52]. Qu et al. found that licorice total flavonoids increased the abundance of *Lactobacillus* in the gut [53]. Consequently, plant extracts, including grapevine leaves extracts, can both inhibit pathogens and promote probiotics. These responses reflect interspecies and interstrain differences in cellular structure, intrinsic susceptibility, and the ability to metabolize or utilize phytochemicals [52,54]. Due to the complexity of the *V. amurensis* extract, clarifying the background of its growth-promoting effect requires further investigation, including the isolation of individual fractions and single phenolic compounds, followed by their microbiological evaluation.

Our microbiological results further demonstrated a pronounced protective effect of the *V. amurensis* extract against H_2_O_2_-induced oxidative stress during the growth of *L. plantarum*. Although the strain was still able to grow in the presence of 1 mM H_2_O_2_, its growth rate was reduced compared with the unstressed control (Figure 6; Table 1). At 2 mM H_2_O_2_, no bacterial growth was detected in the absence of the extract, whereas extract-supplemented cultures maintained substantial growth and reached a viable cell count of 1.8 × 10^8^ CFU mL^−1^ after 24 h. Under more severe oxidative stress, no viable cells were detected after 24 h at 5 or 10 mM H_2_O_2_ in the absence of the extract, whereas the extract maintained a quantifiable viable population at 5 mM H_2_O_2_. At 10 mM H_2_O_2_, only sporadic colonies were recovered and the viable cell number could not be reliably quantified.

Importantly, the protective effect observed under H_2_O_2_-induced oxidative stress cannot be attributed solely to a simple direct interaction between the extract and H_2_O_2_. Following a prolonged lag phase, cultures supplemented with the extract maintained substantial growth under oxidative stress, reaching final OD_630_ values and viable cell numbers that exceeded those of the unstressed control. At 1 mM H_2_O_2_, the maximum growth rate and AUC also markedly exceeded the corresponding values of the unstressed control. These findings are consistent with the growth-promoting effect of the extract observed in the absence of H_2_O_2_. Thus, although direct scavenging of H_2_O_2_ by extract constituents may contribute to the observed protection, the growth response suggests that additional effects on bacterial growth and/or stress adaptation are also involved.

According to the literature data, H_2_O_2_ tolerance in *L. plantarum* is probably highly strain-dependent, and the tolerance of our isolated strain appears to be relatively low. For instance, *L. plantarum* KM1 as well as the GXL94 strain were capable of growing even in the presence of 5 mM H_2_O_2_ [55,56]. This oxidative stress-mitigating property of the extract could be crucial for human applications where probiotics need to be protected from oxidative environments, particularly against reactive oxygen species.

In summary, the present microbiological results provide preliminary evidence that the polyphenol-rich *V. amurensis* leaf extract promoted the growth of *L. plantarum* and enhanced its survival under H_2_O_2_-induced oxidative stress. However, the microbiological experiments were performed *in vitro* using a single *L. plantarum* isolate. Therefore, these findings are strain-specific and require validation using additional bacterial strains and more complex biological systems before potential functional applications can be considered. Furthermore, the mechanisms underlying the observed growth-promoting and protective effects were not investigated directly and remain to be elucidated. Nevertheless, the high disease resistance of *V. amurensis* may reduce the need for intensive plant protection, making this species an attractive candidate for the sustainable production of grape leaf-derived bioactive compounds.

## 5. Conclusions

Flavonols together with a hydroxycinnamic acid derivative, caftaric acid, represent the major phenolic constituents of grapevine leaves in both the investigated wild *Vitis* species and the *V. vinifera* cultivars. Although the main flavonols predominantly consist of the glycosylated forms of quercetin and kaempferol, the diverse glycosylation of these two aglycones, as well as the concentration of individual compounds, creates a unique, genotype-dependent phenolic pattern. Notably, the concentrations of different phenolic compounds differed by as much as two orders of magnitude among the investigated genotypes, highlighting grapevine leaves as a remarkably diverse natural source of bioactive phenolics. These findings provide valuable insights for selecting grapevine genotypes enriched in specific phenolic compounds for future phytochemical, biotechnological, and nutritional research. Demonstrating the biological relevance of this phytochemical diversity, an aqueous leaf extract of the phenolic-rich *V. amurensis* was evaluated using the probiotic bacterium as a model organism. The extract significantly promoted bacterial growth, mitigated the effects of H_2_O_2_-induced oxidative stress, and enhanced bacterial survival even under severe oxidative stress conditions. These preliminary findings were obtained under *in vitro* conditions using a single *L. plantarum* isolate and therefore require validation using additional bacterial strains and more complex biological systems. Overall, grapevine leaves represent a promising and sustainable source of bioactive phenolic compounds with considerable potential, warranting further investigations for various functional applications.

## Figures and Tables

**Table 1:** Growth kinetic parameters of *L. plantarum* cultured with different concentrations of *V. amurensis* leaf extract and H_2_O_2_.

Condition	H_2_O_2_ (mM)	Extract (mg mL^−1^)	Max Growth Rate (h^−1^)	Lag Time (h)	AUC (OD_630_ × h)	OD_630_ at 24 h	Viable Cell Count at 24 h (cfu mL^−1^)
Non-stressed	0	0	0.0284 ± 0.0019	1.62 ± 0.07	3.32 ± 0.11	0.17 ± 0.00	8.3 × 10^7^ ± 6.0 × 10^6^
	0	0.11	0.0303 ± 0.0013	1.79 ± 0.04	3.49 ± 0.10	0.17 ± 0.01	1.2 × 10^8^ ± 5.8 × 10^6^ **
	0	0.55	0.0382 ± 0.0003 ***	1.96 ± 0.17 *	4.26 ± 0.11 ***	0.20 ± 0.00 ***	1.5 × 10^8^ ± 2.1 × 10^7^ ***
	0	2.75	0.0649 ± 0.0007 ***	2.20 ± 0.14 ***	6.69 ± 0.31 ***	0.34 ± 0.01 ***	2.0 × 10^8^ ± 7.6 × 10^6^ ***
	0	5.50	0.0682 ± 0.0007 ***	2.28 ± 0.06 ***	8.55 ± 0.19 ***	0.48 ± 0.01 ***	2.4 × 10^8^ ± 3.2 × 10^7^ ***
H_2_O_2_-stressed	1	0	0.0264 ± 0.0017	1.97 ± 0.06	3.15 ± 0.23	0.19 ± 0.03	6.5 × 10^7^ ± 5.0 × 10^6^
	2	0	n.a.	n.a.	-	-	n.d.
	5	0	n.a.	n.a.	-	-	n.d.
	10	0	n.a.	n.a.	-	-	n.d.
	1	5.50	0.0593 ± 0.0008 ***	3.37 ± 0.07 ***	6.20 ± 0.84 *	0.35 ± 0.04 **	2.0 × 10^8^ ± 2.1 × 10^7^ ***
	2	5.50	0.0304 ± 0.0005	4.97 ± 0.71	4.06 ± 0.26	0.34 ± 0.02	1.8 × 10^8^ ± 2.3 × 10^7^
	5	5.50	n.a.	n.a.	-	-	5.5 × 10^2^ ± 5.0 × 10^1^
	10	5.50	n.a.	n.a.	-	-	n.q. (<100)

n.a., not applicable; kinetic parameters could not be determined because no evaluable growth curve was observed under these conditions. n.d., not detected (<10 CFU mL^−1^); n.q., detected but below the limit of quantification (100 CFU mL^−1^). For non-stressed cultures, statistical significance was determined using one-way ANOVA followed by Dunnett’s multiple-comparison test against the untreated control. For H_2_O_2_-stressed cultures, corresponding treatments with and without the extract were compared using Welch’s independent-samples t-test where quantitative values were available for both treatments. * *p* < 0.05, ** *p* < 0.01, *** *p* < 0.001.

## Data Availability

The original contributions presented in this study are included in the article/Supplementary Materials. Further inquiries can be directed to the corresponding author.

## Supplementary Materials

The following supporting information can be downloaded at https://www.mdpi.com/article/10.3390/antiox15101266/s1, Table S1. Analyzed grapevine accessions and genotypic data for nine SSR loci; Table S2. Calibration and validation parameters of the HPLC-DAD method for phenolic compounds; Table S3. Composition of the culture media used for *L. plantarum* growth and background measurements; Table S4. Pairwise comparisons of total phenolic content among grapevine genotypes using the Games–Howell post hoc test; Table S5. Pairwise comparisons of caftaric acid among grapevine genotypes using the Games–Howell post hoc test; Table S6. Pairwise comparisons of quercetin-3-*O*-rutinoside among grapevine genotypes using the Games–Howell post hoc test; Table S7. Pairwise comparisons of quercetin-3-*O*-galactoside among grapevine genotypes using the Games–Howell post hoc test; Table S8. Pairwise comparisons of quercetin-3-*O*-glucoside among grapevine genotypes using the Games–Howell post hoc test; Table S9. Pairwise comparisons of quercetin-3-*O*-glucuronide among grapevine genotypes using the Games–Howell post hoc test; Table S10. Pairwise comparisons of kaempferol-3-*O*-rutinoside among grapevine genotypes using the Games–Howell post hoc test; Table S11. Pairwise comparisons of kaempferol-3-*O*-glucoside among grapevine genotypes using the Games–Howell post hoc test; Table S12. Pairwise comparisons of kaempferol-3-*O*-glucuronide among grapevine genotypes using the Games–Howell post hoc test; Table S13. Final concentrations of the crude extract and individual phenolic compounds in LB medium used in the *L. plantarum* growth experiments.

## Abbreviations

The following abbreviations are used in this manuscript:

AUC	area under the curve
CA	caftaric acid
CFU	colony-forming units
DW	dry weight
FW	fresh weight
GAE	gallic acid equivalents
HCA	hierarchical cluster analysis
HPLC-DAD	high-performance liquid chromatography with diode-array detection
K-glc	kaempferol-3-*O*-glucoside
K-glr	kaempferol-3-*O*-glucuronide
K-rut	kaempferol-3-*O*-rutinoside
LB	lysogeny broth
LOD	limit of detection
LOQ	limit of quantification
OD_630_	optical density at 630 nm
Q-gal	quercetin-3-*O*-galactoside
Q-glc	quercetin-3-*O*-glucoside
Q-glr	quercetin-3-*O*-glucuronide
Q-rut	quercetin-3-*O*-rutinoside
RSD	relative standard deviation
TPC	total phenolic content
