# Supplementary material for: Phenolic Profiling of Grapevine Leaves and the Protective Effect of a Polyphenol-Rich Vitis amurensis Extract on Lactiplantibacillus plantarum Under Oxidative Stress
Source: Antioxidants (Basel). 2026 Oct 1;15(10):1266. doi: 10.3390/antiox15101266 (PMC13700809; doi:10.3390/antiox15101266)
Supplement: Supplementary file 1 [file antioxidants-15-01266-s001.zip › antioxidants-4542454-supplementary.pdf]

Supplementary Materials

Phenolic profiling of grapevine leaves and the protective effect of a polyphenol-rich *Vitis amurensis* extract on *Lactiplantibacillus plantarum* under oxidative stress

Table S1. Analyzed grapevine accessions and genotypic data for nine SSR loci.

Prime name	Variety number *V* IVC	VVMD27:1	VVMD27:2	VVS2:1	VVS2:2	VVMD7:1	VVMD7:2	VVMD5:1	VVMD5:2	VRZAG62:1	VRZAG62:2	VRZAG79:1	VRZAG79:2	VVMD28:1	VVMD28:2	VVMD32:1	VVMD32:2	VVMD25:1	VVMD25:2
V. amurensis	-	188	190	139	141	241	245	236	240	186	188	245	257	248	252	238	248	237	237
V. coignetiae	-	184	188	133	139	239	251	236	242	187	193	239	239	234	234	238	238	239	243
V. davidii	-	178	192	133	141	237	239	242	244	184	188	241	241	226	258	232	242	237	247
V. pentagona	-	186	195	129	171	237	238	236	250	174	196	253	261	238	242	242	242	249	249
V. piasezkii	-	184	190	133	157	239	249	242	248	194	196	251	255	248	264	238	240	235	241
Riparia Gloire de Montpellier	4824	208	212	141	145	251	265	268	268	192	200	255	259	216	244	md	md	237	239
V. romanetii	-	184	186	129	129	243	245	248	250	214	214	247	249	220	238	248	248	251	255
Rupestris du Lot	10389	206	208	137	137	257	261	238	270	196	196	259	263	220	242	235	237	237	237
V. sylvestris	-	184	190	133	135	239	247	236	238	190	194	251	251	236	240	250	272	239	249
Dabouki	3309	180	184	135	151	247	249	236	238	188	204	247	247	258	258	250	272	241	245
Furmint	4292	180	195	133	153	239	249	228	242	188	204	237	249	228	248	264	272	239	241
Kishmish vatkana	6277	180	195	137	145	239	249	236	242	188	202	247	259	218	234	250	272	239	239
Manseng petit blanc	7339	190	190	151	155	239	257	234	240	194	204	247	251	234	234	240	272	239	249
Pinot noir	9279	186	190	137	151	239	243	230	240	188	194	239	245	218	236	240	272	239	249
Sárga muskotály	8193	180	195	133	133	233	249	230	238	186	196	251	255	246	268	264	272	241	249
Tannat	12257	186	190	143	155	249	249	240	242	194	200	239	251	234	244	240	256	255	255
Isabella	5560	180	184	123	151	235	249	240	240	202	204	237	247	226	236	248	272	241	249

Table S2. Calibration and validation parameters of the HPLC-DAD method for phenolic compounds.

Phenolic compound	Calibration range (µg/mL)	Calibration equation	R^2	[a]LOD (µg/mL)	[a]LOQ (µg/mL)	[b]Intra-day precision (RSD, %)	[c]Inter-day precision (RSD, %)	[d]Method repeatability (RSD, %)	[e]Recovery (%)
Caftaric acid	2.5 - 250	y = 19109.66 – 23174.07	0.999959	0.6	1.7	0.42	0.54	0.7	97.7 ± 2.1
Quercetin-3-*O*-rutinoside	2.0 - 200	y = 8556.85 –37.93	0.999991	0.3	1.0	0.49	0.58	1.6	101.2 ± 1.6
Quercetin-3-*O*-galactoside	2.1 - 210	y = 12058.81 – 951.92	0.999990	0.4	1.2	0.50	0.50	1.3	102.5 ± 3.0
Quercetin-3-*O*-glucoside	2.1 - 210	y = 10776.37 – 968.00	0.999992	0.4	1.1	0.33	0.54	1.2	97.0 ± 3.4
Quercetin-3-*O*-glucuronide	2.0 - 200	y = 10824.11 – 3019.81	0.999992	0.5	1.4	0.39	0.69	0.8	98.3 ± 3.2
Kaempferol-3-*O*-rutinoside	2.0 - 200	y = 8139.10 – 364.99	0.999985	0.4	1.1	0.31	0.57	0.8	101.6 ± 4.1
Kaempferol-3-*O*-glucoside	2.1 - 210	y = 12052.09 – 363.34	0.999984	0.5	1.4	0.29	0.56	1.3	102.0 ± 4.8
Kaempferol-3-*O*-glucuronide	2.1 - 210	y = 11222.52 – 264.63	0.999984	0.3	1.1	0.69	0.82	1.3	101.6 ± 2.4

[a] The limits of detection (LOD) and quantification (LOQ) were estimated according to LOD = 3.3σ/S and LOQ = 10 σ/S, where σ is the standard deviation of the response, estimated as the residual standard deviation of the regression.
[b] Intra-day precision was evaluated from replicate measurements of the standard mixture performed within the same day (n = 5) and was expressed as relative standard deviation (RSD).
[c] Inter-day precision was evaluated by measuring the standard mixture on three consecutive days (n = 3) and was expressed as relative standard deviation (RSD).
[d] Method repeatability was evaluated using three independently prepared and extracted aliquots of the same leaf sample (n = 3) and was expressed as relative standard deviation (RSD).
[e] $\text{Recovery (\%)} = ((c_{spiked} - c_{unspiked})/c_{added}) \times 100$. Samples were spiked prior to extraction at three concentration levels (n = 3 per level), and recovery is reported as the overall mean ± SD (n = 9).

Table S3. Composition of the culture media used for *L. plantarum* growth and background measurements.

ID	LB with *L. plantarum*	LB without *L. plantarum*	H_2O	200 mM H_2O_2	110 mg mL^{-1} extract
	µl				
Control	900	0	100	0	0
Control background	0	900	100	0	0
E1	900	0	99	0	1
E1 background	0	900	99	0	1
E5	900	0	95	0	5
E5 background	0	900	95	0	5
E25	900	0	75	0	25
E25 background	0	900	75	0	25
E50	900	0	50	0	50
E50 background	0	900	50	0	50
H1E50	900	0	45	5	50
H1E50 background	0	900	45	5	50
H2E50	900	0	40	10	50
H2E50 background	0	900	40	10	50
H5E50	900	0	25	25	50
H5E50 background	0	900	25	25	50
H10E50	900	0	0	50	50
H10E50 background	0	900	0	50	50

Table S4. Pairwise comparisons of total phenolic content among grapevine genotypes using the Games–Howell post hoc test.

TPC	*V. amurensis*	Tannat	*V. davidii*	*V. romanetii*	*V. piasezkii*	Manseng petit blanc	*V. pentagona*	Furmint	Sárga muskotály	Riparia gloire	Pinot noir	*V. sylvestris*	Kismish vatkana	Rupestris du Lot	Dabouki	*V. coignetiae*	Isabella
V. amurensis	—																
Tannat	0.317	—															
V. davidii	0.200	0.942	—														
V. romanetii	0.121	0.241	0.995	—													
V. piasezkii	0.116	0.571	0.995	1.000	—												
Manseng petit blanc	0.088	0.299	0.954	1.000	1.000	—											
V. pentagona	0.128	0.093	0.801	0.923	1.000	1.000	—										
Furmint	0.113	**0.049**	0.793	0.935	1.000	1.000	1.000	—									
Sárga muskotály	0.067	0.217	0.797	0.945	1.000	1.000	0.999	1.000	—								
Riparia gloire	0.053	0.152	0.590	0.724	0.976	0.978	0.885	0.939	1.000	—							
Pinot noir	0.091	**0.040**	0.410	0.284	0.833	0.743	**0.004**	0.195	0.982	1.000	—						
V. sylvestris	0.057	**0.009**	0.258	0.118	0.591	0.450	0.140	0.117	0.746	0.965	0.628	—					
Kismish vatkana	0.070	**0.016**	0.251	0.120	0.524	0.383	**0.013**	**0.034**	0.624	0.879	0.079	1.000	—				
Rupestris du Lot	0.052	**0.005**	0.190	0.064	0.422	0.278	0.065	**0.036**	0.480	0.725	0.218	0.955	0.944	—			
Dabouki	**0.023**	**0.029**	0.117	0.093	0.295	0.200	0.181	0.167	0.347	0.540	0.381	0.750	0.764	0.974	—		
V. coignetiae	**0.022**	**0.006**	0.064	**0.020**	0.148	0.070	0.066	**0.040**	0.125	0.193	0.118	0.173	0.213	0.320	0.947	—	
Isabella	**0.022**	**0.004**	0.061	**0.015**	0.137	0.062	0.052	**0.027**	0.108	0.165	0.091	0.118	0.156	0.223	0.871	1.000	—

Data are presented as Games–Howell-adjusted *p*-values following Welch's one-way ANOVA. Significant differences ($p < 0.05$) are indicated in bold and by a color scale; darker shading indicates smaller adjusted *p*-values within the significant range.

Table S5. Pairwise comparisons of caftaric acid among grapevine genotypes using the Games–Howell post hoc test.

CA	*V. amurensis*	Tannat	*V. davidii*	*V. romanetii*	*V. piasezkii*	Manseng petit blanc	*V. pentagona*	Furmint	Sárga muskotály	Riparia gloire	Pinot noir	*V. sylvestris*	Kismish vatkana	Rupestris du Lot	Dabouki	*V. coignetiae*	Isabella
V. amurensis	—																
Tannat	0.386	—															
V. davidii	0.109	**0.003**	—														
V. romanetii	0.270	**0.006**	**0.009**	—													
V. piasezkii	0.985	**0.019**	**0.012**	0.070	—												
Manseng petit blanc	1.000	0.127	**0.016**	**0.048**	0.858	—											
V. pentagona	**0.020**	<0.001	**0.002**	<0.001	<0.001	**0.001**	—										
Furmint	1.000	0.260	**0.039**	0.106	0.889	1.000	**0.005**	—									
Sárga muskotály	0.878	**0.012**	**0.010**	0.091	0.999	0.490	<0.001	0.616	—								
Riparia gloire	1.000	**0.041**	<0.001	<0.001	0.994	0.975	<0.001	0.980	0.544	—							
Pinot noir	1.000	0.068	**0.005**	**0.018**	0.880	1.000	<0.001	1.000	0.417	0.989	—						
V. sylvestris	0.883	**0.012**	0.136	0.618	0.999	0.601	**0.007**	0.665	1.000	0.815	0.613	—					
Kismish vatkana	0.767	1.000	**0.022**	**0.042**	0.174	0.556	**0.005**	0.713	0.118	0.250	0.419	0.105	—				
Rupestris du Lot	0.173	**0.003**	0.435	0.685	**0.023**	**0.023**	<0.001	0.060	**0.023**	<0.001	**0.006**	0.317	**0.028**	—			
Dabouki	0.381	**0.004**	0.798	1.000	0.466	0.130	**0.050**	0.177	0.700	0.218	0.130	0.940	**0.032**	0.999	—		
V. coignetiae	**0.018**	**0.001**	<0.001	<0.001	<0.001	**0.001**	0.977	**0.005**	<0.001	<0.001	<0.001	**0.007**	**0.005**	<0.001	**0.038**	—	
Isabella	**0.019**	<0.001	**0.003**	<0.001	<0.001	**0.001**	1.000	**0.004**	<0.001	<0.001	<0.001	**0.006**	**0.005**	<0.001	**0.045**	0.997	—

Data are presented as Games–Howell-adjusted *p*-values following Welch's one-way ANOVA. Significant differences ($p < 0.05$) are indicated in bold and by a color scale; darker shading indicates smaller adjusted *p*-values within the significant range.

Table S6. Pairwise comparisons of quercetin-3-*O*-rutinoside among grapevine genotypes using the Games–Howell post hoc test.

Q-rut	*V. amurensis*	Tannat	*V. davidii*	*V. romanetii*	*V. piasezkii*	Manseng petit blanc	*V. pentagona*	Furmint	Sárga muskotály	Riparia gloire	Pinot noir	*V. sylvestris*	Kismish vatkana	Rupestris du Lot	Dabouki	*V. coignetiae*	Isabella
V. amurensis	—																
Tannat	<0.001	—															
V. davidii	0.634	<0.001	—														
V. romanetii	**0.002**	<0.001	**0.001**	—													
V. piasezkii	**0.004**	<0.001	**0.004**	0.262	—												
Manseng petit blanc	<0.001	**0.001**	<0.001	<0.001	<0.001	—											
V. pentagona	**0.002**	0.261	**0.002**	**0.003**	**0.003**	**0.008**	—										
Furmint	**0.001**	**0.003**	**0.001**	**0.001**	**0.001**	**0.007**	0.199	—									
Sárga muskotály	<0.001	**0.002**	<0.001	<0.001	<0.001	**0.007**	**0.016**	**0.026**	—								
Riparia gloire	0.361	<0.001	0.129	**0.002**	**0.004**	<0.001	**0.002**	**0.001**	<0.001	—							
Pinot noir	<0.001	0.052	<0.001	<0.001	<0.001	<0.001	**1.000**	0.059	**0.001**	<0.001	—						
V. sylvestris	**0.001**	**0.001**	**0.001**	**0.001**	**0.001**	**0.026**	**0.022**	0.120	0.306	**0.001**	<0.001	—					
Kismish vatkana	<0.001	0.197	<0.001	<0.001	<0.001	<0.001	1.000	**0.007**	<0.001	<0.001	0.705	<0.001	—				
Rupestris du Lot	**0.003**	<0.001	**0.030**	**0.006**	**0.010**	<0.001	**0.002**	**0.001**	<0.001	**0.004**	<0.001	**0.001**	<0.001	—			
Dabouki	**0.008**	0.776	**0.009**	**0.012**	**0.014**	**0.036**	1.000	0.403	0.059	**0.008**	1.000	0.098	1.000	**0.009**	—		
V. coignetiae	**0.027**	<0.001	**0.021**	**0.001**	**0.003**	<0.001	**0.002**	**0.001**	<0.001	0.195	<0.001	**0.001**	<0.001	<0.001	**0.008**	—	
Isabella	<0.001	<0.001	<0.001	0.404	0.060	<0.001	**0.002**	**0.001**	<0.001	<0.001	<0.001	**0.001**	<0.001	**0.002**	**0.011**	<0.001	—

Data are presented as Games–Howell-adjusted *p*-values following Welch's one-way ANOVA. Significant differences ($p < 0.05$) are indicated in bold and by a color scale; darker shading indicates smaller adjusted *p*-values within the significant range.

Table S7. Pairwise comparisons of quercetin-3-*O*-galactoside among grapevine genotypes using the Games–Howell post hoc test.

Q-gal	*V. amurensis*	Tannat	*V. davidii*	*V. romanetii*	*V. piasezkii*	Manseng petit blanc	*V. pentagona*	Furmint	Sárga muskotály	Riparia gloire	Pinot noir	*V. sylvestris*	Kismish vatkana	Rupestris du Lot	Dabouki	*V. coignetiae*	Isabella
V. amurensis	—																
Tannat	0.576	—															
V. davidii	0.373	1.000	—														
V. romanetii	0.856	0.652	0.635	—													
V. piasezkii	0.194	0.855	0.843	0.481	—												
Manseng petit blanc	0.099	0.810	0.632	0.537	0.999	—											
V. pentagona	**0.017**	0.050	**0.011**	0.365	0.939	**0.011**	—										
Furmint	0.168	0.988	0.984	0.562	0.998	1.000	0.106	—									
Sárga muskotály	**0.011**	**0.024**	**0.005**	0.316	0.640	**0.003**	**0.001**	**0.041**	—								
Riparia gloire	**0.012**	**0.032**	**0.005**	0.344	0.839	**0.003**	0.688	0.064	0.317	—							
Pinot noir	0.326	1.000	1.000	0.632	0.817	0.362	**0.001**	0.961	**0.001**	<0.001	—						
V. sylvestris	**0.024**	0.107	**0.020**	0.416	1.000	0.061	0.276	0.317	**0.019**	0.082	**0.002**	—					
Kismish vatkana	**0.042**	0.209	0.062	0.452	1.000	0.227	**0.002**	0.603	<0.001	**0.001**	**0.008**	0.740	—				
Rupestris du Lot	**0.007**	**0.012**	**0.003**	0.274	0.356	**0.001**	<0.001	**0.018**	<0.001	**0.006**	<0.001	**0.004**	<0.001	—			
Dabouki	**0.005**	**0.010**	**0.001**	0.284	0.419	<0.001	**0.012**	**0.015**	0.296	**0.042**	<0.001	**0.002**	<0.001	0.994	—		
V. coignetiae	**0.025**	0.249	0.146	0.440	1.000	0.671	0.697	0.744	0.209	0.449	0.114	1.000	1.000	0.067	0.076	—	
Isabella	**0.006**	**0.010**	**0.002**	0.260	0.283	**0.001**	<0.001	**0.014**	<0.001	**0.003**	<0.001	**0.003**	<0.001	**0.032**	0.470	**0.046**	—

Data are presented as Games–Howell-adjusted *p*-values following Welch's one-way ANOVA. Significant differences ($p < 0.05$) are indicated in bold and by a color scale; darker shading indicates smaller adjusted *p*-values within the significant range.

Table S8. Pairwise comparisons of quercetin-3-*O*-glucoside among grapevine genotypes using the Games–Howell post hoc test.

Q-glc	*V. amurensis*	Tannat	*V. davidii*	*V. romanetii*	*V. piasezkii*	Manseng petit blanc	*V. pentagona*	Furmint	Sárga muskotály	Riparia gloire	Pinot noir	*V. sylvestris*	Kismish vatkana	Rupestris du Lot	Dabouki	*V. coignetiae*	Isabella
V. amurensis	—																
Tannat	0.994	—															
V. davidii	0.056	0.344	—														
V. romanetii	**0.038**	0.218	0.988	—													
V. piasezkii	0.318	0.970	0.206	**0.046**	—												
Manseng petit blanc	0.561	1.000	0.101	**0.031**	0.999	—											
V. pentagona	**0.028**	0.129	0.255	0.589	**0.017**	**0.017**	—										
Furmint	0.259	0.891	0.974	0.742	1.000	0.963	0.361	—									
Sárga muskotály	**0.012**	**0.049**	**0.012**	**0.010**	**0.003**	**0.004**	**0.001**	0.087	—								
Riparia gloire	**0.031**	0.163	0.643	0.996	**0.021**	**0.018**	0.965	0.520	**0.006**	—							
Pinot noir	0.933	1.000	**0.009**	**0.003**	0.471	0.948	**0.003**	0.520	**0.001**	**0.002**	—						
V. sylvestris	**0.039**	0.248	1.000	1.000	0.086	**0.047**	0.621	0.847	**0.027**	0.983	**0.004**	—					
Kismish vatkana	**0.034**	0.174	0.705	0.999	**0.026**	**0.023**	0.789	0.556	**0.002**	1.000	**0.003**	0.994	—				
Rupestris du Lot	**0.007**	**0.029**	**0.003**	**0.001**	**0.001**	**0.002**	<0.001	**0.041**	0.051	<0.001	<0.001	**0.006**	<0.001	—			
Dabouki	**0.005**	**0.029**	**0.004**	**0.008**	<0.001	**0.001**	**0.042**	**0.045**	0.939	**0.017**	<0.001	**0.011**	**0.014**	0.998	—		
V. coignetiae	0.059	0.387	1.000	1.000	0.542	0.315	0.921	0.986	0.233	0.996	0.090	1.000	0.998	0.092	0.130	—	
Isabella	**0.006**	**0.022**	**0.002**	**0.001**	**0.001**	**0.001**	<0.001	**0.028**	**0.001**	<0.001	<0.001	**0.004**	<0.001	0.211	0.466	0.054	—

Data are presented as Games–Howell-adjusted *p*-values following Welch's one-way ANOVA. Significant differences ($p < 0.05$) are indicated in bold and by a color scale; darker shading indicates smaller adjusted *p*-values within the significant range.

Table S9. Pairwise comparisons of quercetin-3-*O*-glucuronide among grapevine genotypes using the Games–Howell post hoc test.

Q-glr	*V. amurensis*	Tannat	*V. davidii*	*V. piasezkii*	Manseng petit blanc	*V. pentagona*	Furmint	Sárga muskotály	Riparia gloire	Pinot noir	*V. sylvestris*	Kismish vatkana	Rupestris du Lot	Dabouki	*V. coignetiae*	Isabella
V. amurensis	—															
Tannat	0.063	—														
V. davidii	**0.004**	<0.001	—													
V. piasezkii	1.000	**0.001**	<0.001	—												
Manseng petit blanc	0.347	0.707	<0.001	0.066	—											
V. pentagona	**0.002**	<0.001	<0.001	<0.001	<0.001	—										
Furmint	0.824	**0.018**	<0.001	0.071	0.655	<0.001	—									
Sárga muskotály	0.999	**0.006**	**0.002**	0.988	0.052	**0.001**	0.198	—								
Riparia gloire	0.182	0.917	<0.001	**0.011**	1.000	<0.001	0.201	**0.020**	—							
Pinot noir	**0.043**	1.000	<0.001	**0.001**	0.495	<0.001	**0.018**	**0.004**	0.709	—						
V. sylvestris	0.441	0.860	**0.001**	0.170	1.000	<0.001	**0.837**	0.100	1.000	0.692	—					
Kismish Vatkana	1.000	**0.001**	<0.001	0.996	**0.036**	<0.001	0.098	1.000	**0.008**	**0.001**	0.096	—				
Rupestris du Lot	0.672	0.110	<0.001	0.113	0.988	<0.001	0.998	0.128	0.707	0.069	0.996	0.072	—			
Dabouki	**0.006**	<0.001	0.095	<0.001	<0.001	**0.007**	<0.001	**0.003**	<0.001	<0.001	<0.001	<0.001	<0.001	—		
V. coignetiae	**0.004**	<0.001	0.319	<0.001	<0.001	**0.005**	<0.001	**0.001**	<0.001	<0.001	<0.001	<0.001	<0.001	0.783	—	
Isabella	**0.004**	<0.001	1.000	<0.001	<0.001	<0.001	<0.001	**0.002**	<0.001	<0.001	**0.001**	<0.001	<0.001	0.114	0.432	—

Data are presented as Games–Howell-adjusted *p*-values following Welch's one-way ANOVA. Significant differences ($p < 0.05$) are indicated in bold and by a color scale; darker shading indicates smaller adjusted *p*-values within the significant range. *V. romanetii* was excluded from the statistical analysis because Q-glr was not detected in any of the five biological replicates, resulting in zero within-group variance.

Table S10. Pairwise comparisons of kaempferol-3-*O*-rutinoside among grapevine genotypes using the Games–Howell post hoc test.

K-rut	*V. amurensis*	Tannat	*V. davidii*	*V. romanetii*	*V. piasezkii*	Manseng petit blanc	*V. pentagona*	Furmint	Sárga muskotály	Riparia gloire	Pinot noir	*V. sylvestris*	Kismish vatkana	Rupestris du Lot	Dabouki	*V. coignetiae*	Isabella
V. amurensis	—																
Tannat	**0.001**	—															
V. davidii	0.164	**0.001**	—														
V. romanetii	1.000	**0.002**	0.190	—													
V. piasezkii	0.542	**0.002**	0.539	0.239	—												
Manseng petit blanc	**0.035**	**0.003**	0.999	**0.006**	0.178	—											
V. pentagona	<0.001	0.089	**0.006**	<0.001	<0.001	<0.001	—										
Furmint	<0.001	1.000	**0.001**	**0.002**	**0.001**	**0.002**	0.092	—									
Sárga muskotály	<0.001	**0.040**	**0.012**	**0.002**	**0.002**	**0.005**	0.969	**0.041**	—								
Riparia gloire	1.000	<0.001	0.189	1.000	0.726	0.087	<0.001	<0.001	<0.001	—							
Pinot noir	<0.001	0.178	**0.002**	<0.001	<0.001	<0.001	**0.949**	0.204	0.674	<0.001	—						
V. sylvestris	<0.001	0.218	**0.003**	<0.001	<0.001	<0.001	0.275	0.246	0.323	<0.001	1.000	—					
Kismish vatkana	<0.001	0.402	<0.001	<0.001	<0.001	<0.001	<0.001	0.240	<0.001	<0.001	<0.001	<0.001	—				
Rupestris du Lot	0.897	**0.002**	0.088	**0.022**	**0.025**	**0.003**	<0.001	**0.001**	**0.002**	0.950	<0.001	<0.001	<0.001	—			
Dabouki	0.050	0.077	0.309	0.062	0.095	0.184	0.944	0.095	1.000	**0.048**	0.763	0.606	**0.016**	**0.044**	—		
V. coignetiae	0.973	**0.001**	0.334	0.940	0.988	0.061	<0.001	**0.001**	**0.001**	0.993	<0.001	<0.001	<0.001	0.141	0.073	—	
Isabella	0.954	**0.002**	0.096	**0.048**	**0.035**	**0.004**	<0.001	**0.001**	**0.002**	0.980	<0.001	<0.001	<0.001	0.967	**0.046**	0.180	—

Data are presented as Games–Howell-adjusted *p*-values following Welch's one-way ANOVA. Significant differences ($p < 0.05$) are indicated in bold and by a color scale; darker shading indicates smaller adjusted *p*-values within the significant range.

Table S11. Pairwise comparisons of kaempferol-3-*O*-glucoside among grapevine genotypes using the Games–Howell post hoc test.

K-glc	*V. amurensis*	Tannat	*V. davidii*	*V. romanetii*	*V. piasezkii*	Manseng petit blanc	*V. pentagona*	Furmint	Sárga muskotály	Riparia gloire	Pinot noir	*V. sylvestris*	Kismish vatkana	Rupestris du Lot	Dabouki	*V. coignetiae*	Isabella
V. amurensis	—																
Tannat	0.276	—															
V. davidii	**0.002**	**0.028**	—														
V. romanetii	<0.001	**0.017**	1.000	—													
V. piasezkii	**0.002**	0.982	**0.005**	<0.001	—												
Manseng petit blanc	**0.031**	0.771	**0.003**	<0.001	0.398	—											
V. pentagona	<0.001	0.163	0.383	**0.034**	<0.001	<0.001	—										
Furmint	0.058	1.000	**0.002**	<0.001	1.000	0.676	**0.003**	—									
Sárga muskotály	0.998	0.371	**0.001**	<0.001	**0.026**	0.287	<0.001	0.114	—								
Riparia gloire	0.420	0.864	**0.001**	<0.001	0.962	1.000	**0.002**	0.930	0.760	—							
Pinot noir	**0.002**	1.000	**0.008**	<0.001	0.178	**0.029**	**0.002**	0.815	**0.003**	0.166	—						
V. sylvestris	**0.005**	0.917	**0.035**	**0.003**	0.062	**0.023**	0.155	0.147	**0.005**	**0.029**	0.597	—					
Kismish vatkana	**0.035**	1.000	**0.005**	<0.001	0.738	0.269	**0.016**	0.993	0.053	0.477	1.000	0.598	—				
Rupestris du Lot	**0.008**	0.082	**0.001**	<0.001	<0.001	<0.001	<0.001	**0.008**	**0.018**	**0.034**	**0.001**	**0.002**	**0.008**	—			
Dabouki	**0.017**	**0.025**	0.169	0.206	**0.027**	**0.023**	0.092	**0.027**	**0.018**	**0.021**	**0.035**	**0.048**	**0.031**	**0.013**	—		
V. coignetiae	0.760	0.785	**0.001**	<0.001	0.878	1.000	**0.003**	0.835	0.971	1.000	0.161	**0.028**	0.393	0.085	**0.019**	—	
Isabella	**0.002**	**0.042**	**0.001**	<0.001	<0.001	<0.001	<0.001	**0.005**	**0.005**	**0.013**	**0.001**	**0.002**	**0.005**	**0.012**	**0.011**	**0.028**	—

Data are presented as Games–Howell-adjusted *p*-values following Welch's one-way ANOVA. Significant differences ($p < 0.05$) are indicated in bold and by a color scale; darker shading indicates smaller adjusted *p*-values within the significant range.

Table S12. Pairwise comparisons of kaempferol-3-*O*-glucuronide among grapevine genotypes using the Games–Howell post hoc test.

K-glr	*V. amurensis*	Tannat	*V. davidii*	*V. piasezkii*	Manseng petit blanc	*V. pentagona*	Furmint	Sárga muskotály	Riparia gloire	Pinot noir	*V. sylvestris*	Kismish vatkana	Rupestris du Lot	Dabouki	*V. coignetiae*	Isabella
V. amurensis	—															
Tannat	**0.003**	—														
V. davidii	<0.001	<0.001	—													
V. piasezkii	0.052	0.474	<0.001	—												
Manseng petit blanc	0.922	**0.006**	<0.001	0.113	—											
V. pentagona	0.068	**0.001**	<0.001	**0.004**	**0.002**	—										
Furmint	**0.001**	1.000	**0.001**	0.142	<0.001	<0.001	—									
Sárga muskotály	0.065	1.000	<0.001	0.988	0.150	**0.013**	0.972	—								
Riparia gloire	**0.026**	0.088	1.000	0.056	**0.035**	**0.018**	0.102	0.069	—							
Pinot noir	0.147	**0.044**	**0.001**	0.774	0.083	<0.001	**0.002**	0.505	**0.047**	—						
V. sylvestris	<0.001	0.191	**0.001**	**0.011**	<0.001	<0.001	**0.257**	0.286	0.167	**0.003**	—					
Kismish vatkana	**0.011**	0.858	**0.001**	0.881	<0.001	<0.001	0.158	1.000	0.073	**0.002**	**0.025**	—				
Rupestris du Lot	**0.041**	0.170	**0.001**	1.000	**0.012**	<0.001	**0.010**	0.907	0.056	0.481	**0.004**	0.197	—			
Dabouki	0.431	**0.001**	<0.001	**0.005**	**0.003**	**0.045**	<0.001	**0.022**	**0.022**	<0.001	<0.001	<0.001	<0.001	—		
V. coignetiae	0.948	**0.002**	<0.001	**0.015**	**0.027**	**0.002**	<0.001	**0.041**	**0.026**	<0.001	<0.001	<0.001	<0.001	0.299	—	
Isabella	0.099	**0.001**	<0.001	**0.005**	**0.002**	**0.009**	<0.001	**0.015**	**0.019**	<0.001	<0.001	<0.001	<0.001	0.108	**0.003**	—

Data are presented as Games–Howell-adjusted *p*-values following Welch's one-way ANOVA. Significant differences ($p < 0.05$) are indicated in bold and by a color scale; darker shading indicates smaller adjusted *p*-values within the significant range. *V. romanetii* was excluded from the statistical analysis because K-glr was not detected in any of the five biological replicates, resulting in zero within-group variance.

Table S13. Final concentrations of the crude extract and individual phenolic compounds in LB medium used in the *L. plantarum* growth experiments.

Extract	CA	Q-rut	Q-gal	Q-glc	Q-glr	K-rut	K-glc	K-glr	Total flavonol glycosides
mg mL^{-1}	mg L^{-1}								
0.11	8.1*	0.0*	0.5*	1.2*	8.1*	0.0*	0.0*	0.1*	10.0*
0.55	40.7*	0.1*	2.5*	6.1*	40.6*	0.1*	0.2*	0.3*	49.8*
2.75	203.6*	0.4*	12.3*	30.3*	203.1*	0.3*	1.1*	1.4*	249.0*
5.50	407.1# ± 3.5	0.7# ± 0.0	24.6# ± 0.1	60.6# ± 0.2	406.3# ± 1.8	0.7# ± 0.0	2.2# ± 0.0	2.8# ± 0.0	497.9 ± 2.2

* Concentrations of individual phenolic compounds were calculated from the HPLC-DAD profile determined for the highest extract concentration (5.5 mg mL^{-1}; n = 3).

# Individual phenolic compounds quantified by HPLC-DAD.
